# Controlled numerical generation and reconstruction of off-axis digital holograms with object-specific frequency-domain filtering

**DOI:** 10.1371/journal.pone.0360375

**Published:** 2026-10-22

**Authors:** Junsheng Lu, Xiaolong Shi, Jiaqi Sun, Shuhong Ren, Yanan Zeng

**Affiliations:** 1 School of Life Sciences, Tiangong University, Tianjin, China; 2 College of Engineering and Technology, Tianjin Agricultural University, Tianjin, China; University of Milano-Bicocca, ITALY

## Abstract

Deep-learning-based holographic reconstruction requires numerical data with reliable ground truth for training and quantitative evaluation. Experimental hologram acquisition and pixel-level phase annotation can be difficult to control, making physically defined numerical cases useful as reproducible benchmarks. Here, three analytically specified phase targets—a smooth Gaussian distribution, a four-level step distribution, and a radially structured Siemens Star—are used to generate off-axis digital holograms with known phase ground truth. A frequency-domain filtering procedure is applied before inverse Fourier reconstruction, followed by DCT-based global phase unwrapping. Reconstruction quality is quantified using root mean square error (RMSE), mean absolute error (MAE), correlation coefficient, and profile-based error analysis. The results show that reconstruction errors are comparatively small in smooth regions and increase near abrupt or high-spatial-frequency phase transitions. The three cases are therefore presented as controlled benchmark examples for algorithm verification and baseline comparison, not as a complete substitute for experimental holographic datasets or as a complete training corpus for deep-learning models.

## Introduction

Holography was introduced by Gabor as a method for recording and reconstructing optical wavefront information, and off-axis holography later enabled separation of the zero-order and conjugate terms through a spatial carrier [1, 2]. Digital holography subsequently became an important computational approach for quantitative phase measurement and microscopic imaging [3–5].

Quantitative phase imaging is used in applications including label-free microscopy and biological imaging, where phase provides information that is not directly available from conventional intensity images [6–10]. Phase unwrapping is a key processing step when the recovered phase exceeds the principal interval of the arctangent function, and a range of local and global methods has been developed for digital holography [6]. Experimental holograms are valuable for assessing practical performance, but their ground truth is often difficult to obtain because acquisition conditions, optical aberrations, noise, and sample variability are coupled in the measured data [10, 13, 14].

The objective of this study is therefore narrower than the generation of a complete deep-learning dataset. We establish a reproducible numerical benchmark in which the object phase, carrier frequency, sampling parameters, and reconstruction ground truth are explicitly known. The benchmark is intended to support verification of hologram generation, frequency-domain filtering, phase unwrapping, and reconstruction-error analysis. It can also serve as a controlled pretraining or baseline source for later learning-based studies, while recognizing that a practical deep-learning dataset would require substantially greater diversity and experimentally realistic variability [11, 12].

The simulated cases intentionally omit several experimental factors, including amplitude-phase coupling, speckle, sensor nonuniformity, optical aberration, multiple scattering, and complex three-dimensional specimen structure. These omissions are deliberate because they allow the effect of phase structure and spectral filtering to be isolated. Consequently, the present results should be interpreted as controlled numerical evidence rather than as a claim that the synthetic cases reproduce the full complexity of experimental digital holography [10, 13, 14].

## Materials and methods

### Theoretical basis: Off-axis holography, digital reconstruction and phase reconstruction

#### Off-axis holographic carrier frequency separation model.

Off-axis holography achieves spectral separation by introducing a spatial carrier frequency. The core lies in the optimization of the geometric configuration of the reference light wave and the object light wave. Let the reference light wave be an inclined plane wave, and its complex amplitude expression is:


$$\begin{array}{c} \begin{array}{c} U_{R}(x,y)=A_{R}exp\left[j\text{2}\pi \left(f_{x}x+f_{y}y\right)\right] \end{array} \end{array}$$ (1)

Among them, $f_{x}$ and $f_{y}$ are the spatial frequencies in the x and y directions respectively, which are determined by the tilt angle θ of the reference light, $f_{x}=\frac{sin\theta }{\lambda }$, $f_{y}=\text{0}$. By reasonably selecting the fringe period $p(p=\text{1}/f_{x})$, it can be ensured that the zero – order spectrum, the + 1 – order original image spectrum and the −1 – order conjugate image spectrum are completely separated in the frequency domain [2,3].

In the numerical implementation, the carrier frequency parameter is dynamically generated through the code segment f=1/p, and the reference fringe


$$\begin{array}{c} \begin{array}{c} I_{\text{0}\text{1}}(x)=a_{\text{0}}+b_{\text{0}}cos\left(\text{2}\pi f_{x}\right) \end{array} \end{array}$$ (2)

and the deformed fringe


$$\begin{array}{c} \begin{array}{c} I_{\text{1}\text{1}}(x)=a_{\text{0}}+b_{\text{0}}cos\left(\text{2}\pi f_{x}+\phi (x,y)\right) \end{array} \end{array}$$ (3)

of the object model are constructed. Here, ϕ(x,y) is the object phase modulation term.

The three benchmark cases were assigned different carrier/filtering settings according to the numerical implementations used here. The smooth Gaussian case uses a 40-pixel carrier period and a rectangular +1-order passband; the four-level step case uses a 4-pixel carrier period and a Gaussian soft window; the Siemens Star uses a 3-pixel carrier period and a Gaussian soft window. These parameters are fixed experimental settings of the present simulation and are not proposed as universal classification thresholds.

#### Digital reconstruction.

Digital reconstruction in this study is performed entirely in the Fourier domain. After isolation of the selected diffraction order, the filtered spectrum is inverse-transformed to obtain the complex field from which the wrapped phase is calculated. This is a computational reconstruction step rather than an experimental propagation measurement, and no quantitative claim of nanometre-level accuracy is made here. Digital holographic reconstruction and quantitative phase measurement are well established applications of numerical wavefront processing [3–5].

The present implementation does not use an angular-spectrum propagation stage or weighted least-squares unwrapping. Instead, the filtered +1-order spectrum is shifted to the Fourier origin, inverse-transformed, and combined with the conjugate reference field to obtain the wrapped phase. A DCT-based global Poisson solver is then used for phase unwrapping. This description follows the actual numerical workflow used in the supplied MATLAB implementations.

#### Phase reconstruction.

The core bottleneck of phase reconstruction lies in the phase wrapping phenomenon: limited by the periodicity of the arctangent function, the directly reconstructed phase is confined to the interval [-π, π], forming a discontinuous wrapped phase. Phase unwrapping algorithms are required to estimate the 2π integer multiple jumps to recover the real phase.

Global least-squares phase unwrapping formulates phase recovery as a Poisson problem and can be solved efficiently in transform domains; phase-unwrapping strategies for digital holography have been reviewed extensively [6]. In the present work, a DCT-domain solution is used because it provides a deterministic global reconstruction for the noise-free benchmark cases.

The reconstruction procedure therefore consists of Fourier-domain order selection, inverse Fourier transformation, wrapped-phase extraction, gradient correction, divergence-field construction, DCT-domain Poisson solving, and removal of the residual global offset or fitted background. No additional propagation, denoising, or learned post-processing is applied.

### Core algorithm: Adaptive filtering

To examine how spectral bandwidth affects reconstruction, the study compares a rectangular frequency-domain window for the Gaussian phase target with Gaussian soft windows for the four-level step and Siemens Star targets. The purpose is to retain the selected +1-order spectrum while reducing unnecessary spectral truncation. The procedure is benchmark-specific and is not presented as a universal adaptive classifier.

For each benchmark, the + 1-order spectral region is selected using the fixed coordinates and window parameters specified in the corresponding MATLAB implementation. The filtered spectrum is then shifted to the Fourier origin before inverse transformation. The resulting filtering behavior is illustrated in Fig 2.

1. For the present benchmark implementation, a narrowband spectrum with concentrated energy is assigned to the Gaussian phase case and processed using a rectangular window.
2. A broadened spectrum associated with the discontinuous step phase case is processed using a Gaussian soft window. The present thresholds are empirical parameters of the numerical experiment and should not be interpreted as universal object-classification criteria.

Within the tested numerical cases, the selection and filtering steps are executed automatically according to the predefined spectral criteria.

For the Gaussian case, a rectangular window is used to retain the selected spectral region. The window dimensions are fixed in the present simulation, so the method should be described as object-specific filtering rather than an automatically optimized window-size algorithm.

For discontinuous phase objects with stepped mutations, dynamic Gaussian window filtering is applied. The global maximum position of the two-dimensional spectral matrix is detected to locate the zero-frequency coordinate $\left(m_{\text{0}},n_{\text{0}}\right)$ and fundamental frequency center $\left(m_{\text{1}},n_{\text{1}}\right)$. The standard deviation of the Gaussian window is set as $D_{\text{0}}=(n_{\text{1}}-n_{\text{0}})/\text{4}$, and the window function is defined as:


$$\begin{array}{c} \begin{array}{c} \text{filter}(i,j)=exp\left(-\frac{(i-m_{\text{1}}{)}^{\text{2}}+(j-n_{\text{1}}{)}^{\text{2}}}{\text{2}D_{\text{0}}^{\text{2}}}\right) \end{array} \end{array}$$ (4)

After filtering, the selected spectral order is translated to the Fourier origin before inverse transformation. This operation removes the carrier-frequency displacement from the selected order and allows the recovered complex field to be processed without an additional numerical carrier-removal step.


$$\begin{array}{c} \begin{array}{c} U_{f^{\prime }}(u,v)=U_{f}\left(u-f_{x},v-f_{y}\right)\cdot exp\left[-j\text{2}\pi \left(f_{x}x+f_{y}y\right)\right] \end{array} \end{array}$$ (5)

The frequency-domain shift is therefore part of the reconstruction pipeline rather than an independently validated optical correction. Its role in the present work is to place the selected diffraction order at the zero-frequency location before inverse Fourier transformation.

### Experimental process and object modeling

#### Basic sampling and optical parameter setting.

To balance simulation efficiency and spectral resolution, and match the parameters of commercial CCD sensors, the total sampling point is set as N=512 and the physical width of the hologram as L0=4mm. The physical size of a single pixel is calculated as Δx=0.0078125mm. This parameter satisfies the Nyquist sampling theorem, and the maximum measurable spatial frequency of the system is $f_{\text{max}}=\text{1}/(\text{2}\Delta x)=\text{64}\;\text{lp}/\text{mm}$, which covers all high-frequency components of the tested phase objects and avoids spectral aliasing.

Optical parameters refer to conventional laboratory off-axis holography configurations: DC bias a0=0.5,modulation contrast b0=0.5. This combination can make the modulation depth of the holographic interference fringes at the optimal position in the linear response range of the CCD device, A He-Ne laser with wavelength λ=632.8nm is selected as the light source, whose coherence length is more than 20 cm and meets the coherence requirements of holographic interference. For stepped objects, modulation parameters l=300mm and k=100 are set to match the magnification of actual optical paths.

#### Gaussian phase model.

Constructed using the phase distribution function:


$$\begin{array}{c} \begin{array}{c} \phi (x,y)=\text{1}\text{0}exp\left(-x^{\text{2}}-y^{\text{2}}\right) \end{array} \end{array}$$ (6)

where ∇ϕ is phase gradient.

Under paraxial approximation, the model satisfies the constraint$|\nabla \phi |\ll \frac{\text{2}\pi }{\lambda }$.With curvature radius σ=50pixels and λ=632.8nm, the maximum phase gradient is calculated as $|\nabla \phi {|}_{\text{max}}=\phi_{\text{0}}/\sigma \;\approx \text{0}.\text{04}\;\text{rad}/\mu m$, while $k=\text{2}\pi /\lambda \approx \text{9}.\text{93}\;\mu m^{-\text{1}}$, $|\nabla \phi {|}_{\text{max}}\ll k$,ensuringstrict adherence to the paraxial condition.

Maximum phase dynamic range is [0,10]rad, corresponding to height modulation $h_{\text{max}}=\lambda \phi /(\text{4}\pi )=\text{1}.\text{6}\;\mu m$. This parameter setting suppresses multiple scattering and ensures the validity of paraxial approximation. The constructed Gaussian object model is shown in Fig 1.

**Fig 1:**
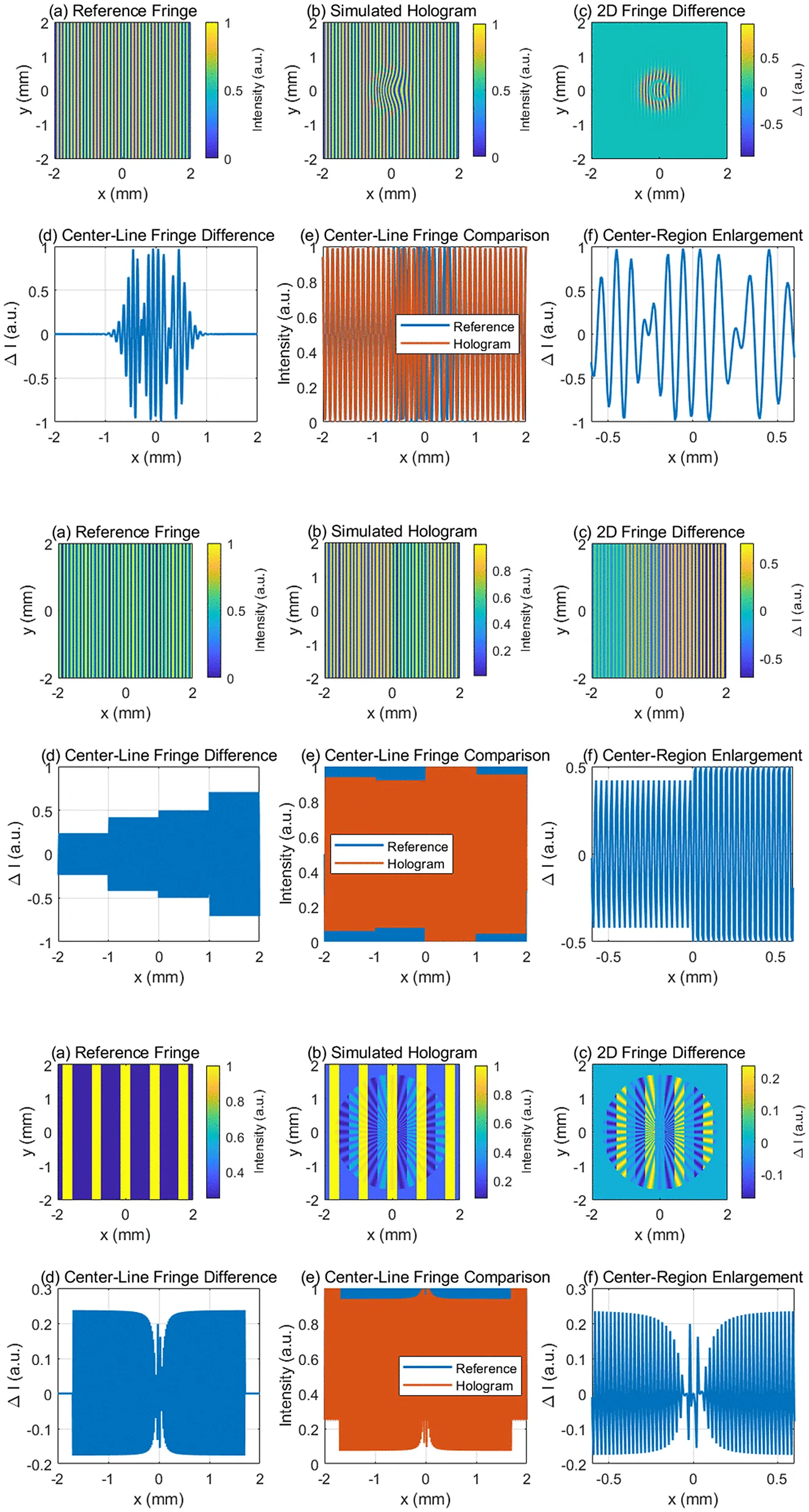
Simulated holograms and fringe modulation for the three benchmark phase targets. (a) Gaussian phase case; (b) four-level step case; (c) Siemens Star case. Each source panel contains the reference fringe, phase-modulated hologram, representative center-line comparison, center-line difference, two-dimensional fringe difference, and local center-region view. Spatial coordinates are reported in mm for the Gaussian and step cases and are mapped to the common image-plane convention for the Siemens Star; intensity is reported in a.u.

#### Step-type phase model.

$\Delta \phi_{\text{step}}$ A four-level discontinuous phase model is constructed with phase values of 0.5, 1.0, 1.5, and 2.0 rad. Each level occupies 128 pixels in the horizontal direction. The model is intended to isolate the effect of one-dimensional phase discontinuities and is not claimed to represent the full morphology of MEMS or microfluidic specimens.

With a 4 mm computational width and 512 samples, each pixel corresponds to approximately 0.0078125 mm (7.8125 μm). Each 128-pixel step therefore spans 1 mm in the numerical field. The phase increment between adjacent levels is 0.5 rad.


$$\begin{array}{c} \begin{array}{c} \Delta x_{\text{fringe}}=\frac{\lambda z}{\text{2}\pi }\Delta \phi_{\text{step}}=\text{6}\text{3}\text{2}.\text{8}\text{nm}\times \;\frac{\text{1}\text{0}\text{0}}{\text{2}\pi }\times \text{0}.\text{5}\approx \text{5}.\text{0357}\;\mu m \end{array} \end{array}$$ (7)

In this experiment, the pixel size is $\Delta x_{\text{pixel}}=\text{7}.\text{8125}\mu m$, which satisfies $\Delta x_{\text{fringe}}<\Delta x_{\text{pixel}}$. This sub-pixel phase jump setting verifies the ability of the adaptive filtering algorithm to retain high-frequency step components and suppress ringing artifacts.

$\Delta x=\frac{p\Delta \phi }{\text{2}\pi }=\text{1}.\text{6}\;\text{pixel},\nabla \phi =\text{0}.\text{5}\;\text{rad}/\text{pixel}$.The step model is consequently used to test sensitivity to abrupt phase transitions rather than to claim that the simulation reproduces a particular industrial device. The expected reconstruction error is assessed directly against the known phase ground truth.

The Gaussian and four-level step targets are complementary controlled cases: the former contains a smooth spatial phase variation, whereas the latter contains abrupt one-dimensional phase transitions. Their simplicity is intentional because it makes the reconstruction error directly traceable to the numerical filtering and unwrapping procedure.

#### Siemens star model.

The Siemens Star is included as a structured phase target with radial spatial-frequency variation. Spoke-pattern targets are widely used to assess resolution in coherent imaging because they sample a broad range of spatial frequencies and orientations within one target [9]. In the present study, the Siemens Star is simulated rather than experimentally fabricated, so its role is to provide a more spatially distributed high-frequency phase structure for numerical verification.

Together, the Gaussian, four-level step, and Siemens Star cases provide three controlled levels of structural complexity: smooth continuous variation, one-dimensional discontinuities, and radially distributed high-frequency boundaries. These cases broaden the numerical benchmark without implying that they statistically represent industrial or biomedical specimens.

### Hologram generation

As shown in (2)(3).The object phase is encoded into a complex phase-only field and interfered numerically with a tilted reference wave to generate the off-axis hologram. The reference and phase-modulated fringes use identical sampling conditions. The resulting intensity distributions are then processed by Fourier-domain filtering and phase reconstruction. Representative hologram and fringe-modulation results are shown in Fig 1.

The Siemens Star is used here as a numerical structured target rather than as a calibrated metrology artifact. Experimental coherent-imaging studies have used Siemens Star targets to characterize resolution and phase-imaging performance [9]. The present simulation does not make an experimental resolution claim and does not use AFM measurements for calibration.

1. AFM-based calibration is not performed in the present study. Such calibration would require an experimentally fabricated reference target and independently measured topography, which are outside the scope of the current numerical benchmark.
2. Similarly, no experimental measurement of detector modulation transfer function or optical aberration is performed. The present frequency-domain analysis evaluates the numerical filtering behavior against analytically known phase ground truth.
3. The fixed phase transitions of the step model and the radial frequency variation of the Siemens Star are used to examine reconstruction sensitivity to phase discontinuities and high-frequency structure. These tests complement the smooth Gaussian benchmark.

### Hologram processing

For each hologram, a two-dimensional Fourier transform is calculated and the selected +1-order spectrum is isolated with the predefined window. The Gaussian case uses a rectangular window, whereas the four-level step and Siemens Star use Gaussian soft windows. The filtered spectra are shown in Fig 2. The filtering is benchmark-specific and is not trained or optimized from the test data.

**Fig 2:**
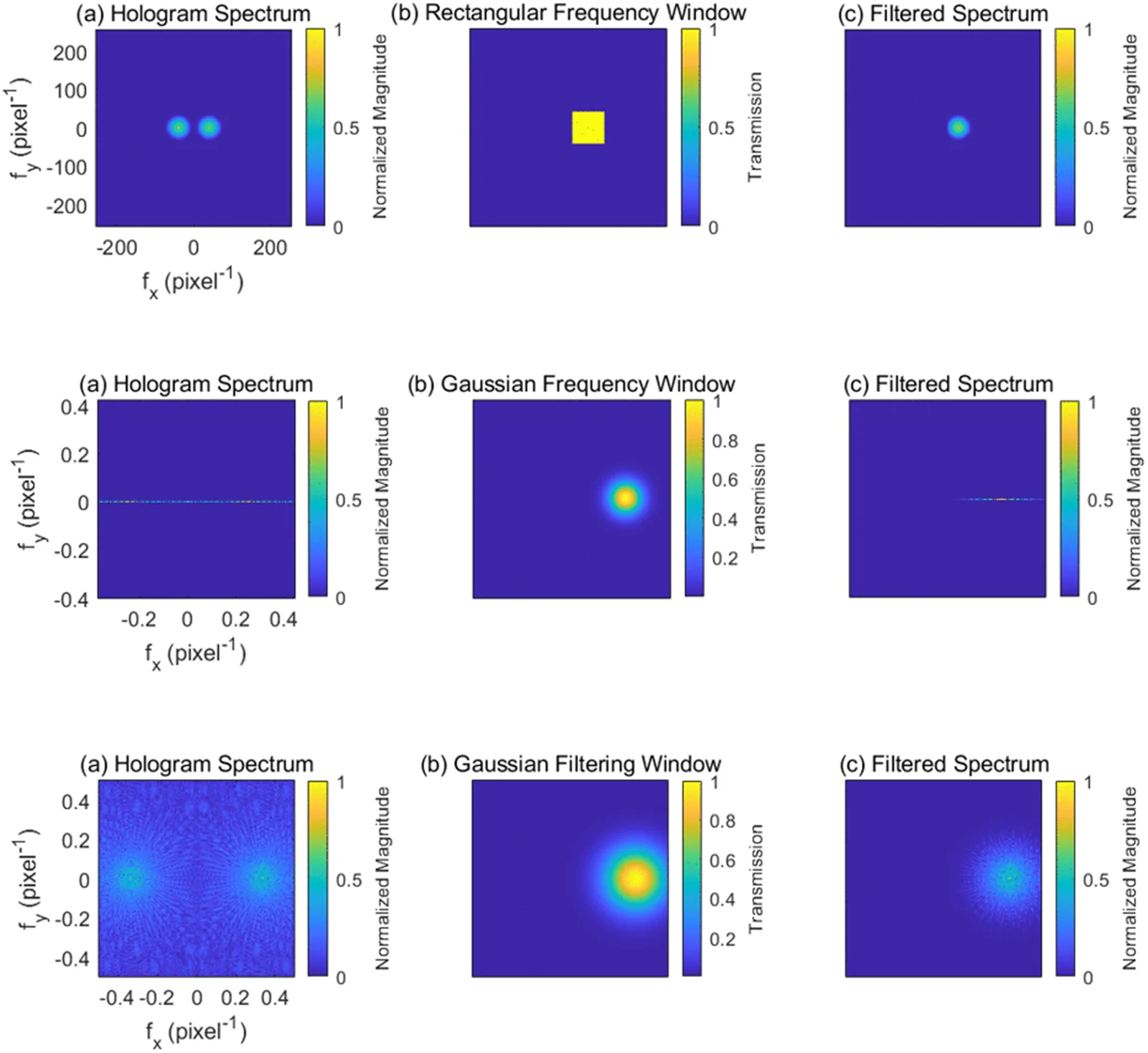
Frequency-domain filtering results for the three benchmark phase targets. The panels show the hologram spectrum, the prescribed frequency-domain window, and the filtered spectrum for each case. Frequency coordinates are reported in pixel−1 and displayed magnitudes are normalized for visualization.

$\Delta \phi =arg(U_{\text{1}})-arg(U_{\text{0}}),U_{\text{1}},U_{\text{0}}$The filtered spectrum is inverse-transformed to obtain the complex field. The wrapped phase is calculated from the product of the reconstructed field and the conjugate reference field. The resulting phase maps for the three benchmark targets are compared in Fig 3.

**Fig 3:**
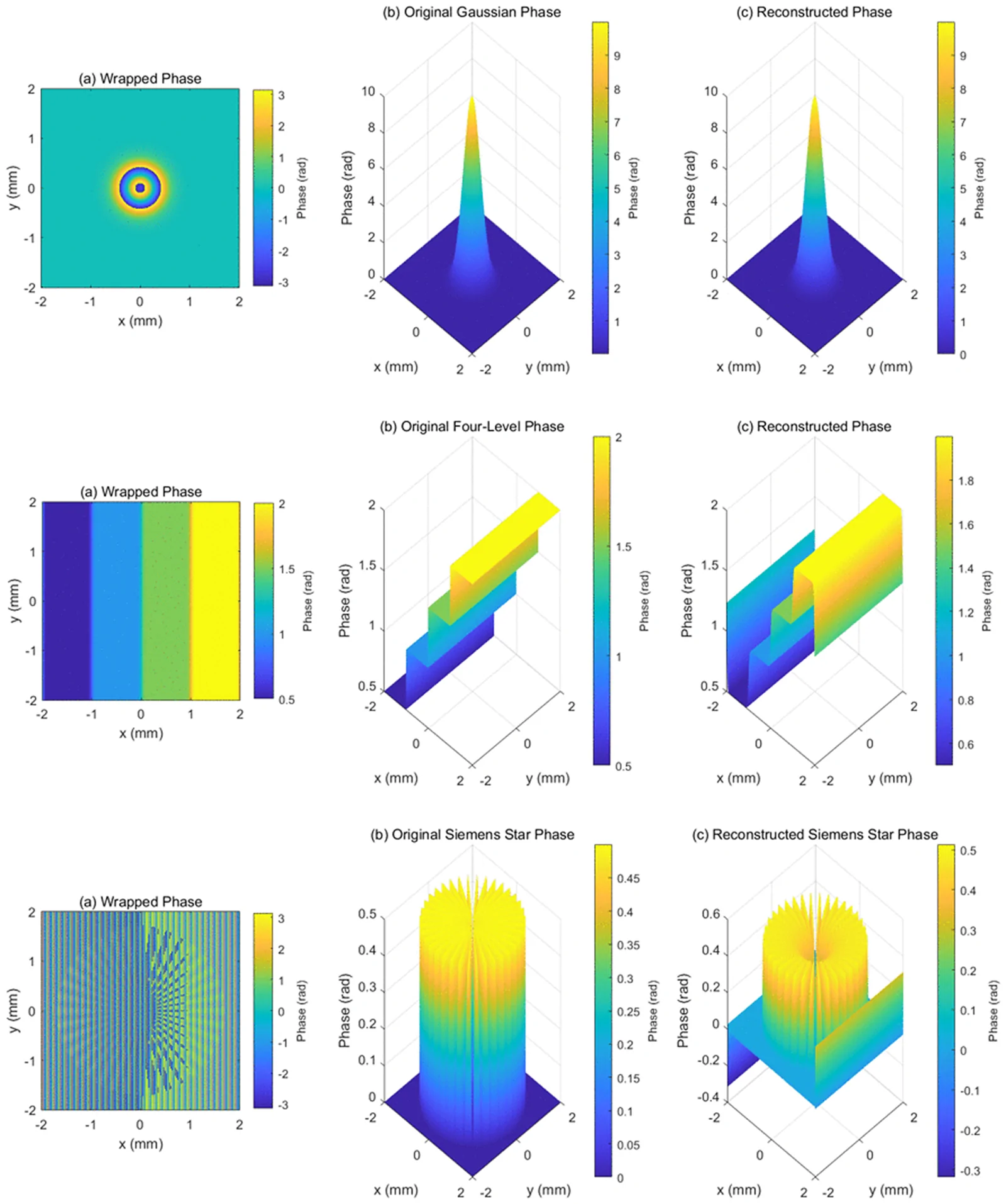
Phase reconstruction results. The panels compare the wrapped phase, analytical phase, and reconstructed phase for the Gaussian, four-level step, and Siemens Star targets. Spatial coordinates are reported in mm and phase is reported in rad.

### DCT-based global phase unwrapping

A DCT-based global phase-unwrapping procedure is used to solve the Poisson equation associated with the corrected wrapped-phase gradient field. Transform-domain phase unwrapping is consistent with the global formulation reviewed for digital holography ^[6]^.


$$\begin{array}{c} \begin{array}{c} \phi_{\text{unwrapped}}=\text{idct2}\left(\frac{\text{dct2}(\nabla^{\text{2}}\phi_{\text{wrapped}})}{\text{2}\left[cos\left(\frac{\pi (m-\text{1})}{M}\right)+cos\left(\frac{\pi (n-\text{1})}{N}\right)-\text{2}\right]+\epsilon }\right) \end{array} \end{array}$$ (8)

where $\nabla^{\text{2}}\phi_{\text{wrapped}}$ is the divergence field of the wrapped phase gradient, and $\epsilon =\;{\text{10}}^{-6}$ is used to avoid division-by-zero errors.

Algorithm Steps:

1. (−π,π]First, horizontal and vertical wrapped-phase gradients are calculated and mapped to the principal interval using atan2(sin(x), cos(x)).
2. Second, the corrected gradients are combined into a divergence field. Neumann boundary conditions are imposed directly at the image boundaries. No separate quality-guided or learned phase-unwrapping stage is used.
3. Third, the divergence field is transformed by the two-dimensional DCT, the Poisson equation is solved in the transform domain, and the inverse DCT yields the continuous phase estimate.

$O(N^{\text{2}}logN)$The DCT step provides a deterministic global solution for the present benchmark. Because the simulations are noise-free, the results should not be interpreted as evidence of noise-suppression performance. Noise robustness remains a separate experimental question [13].

In this experiment, the wrapped phase gradient calculation of Gaussian object and step object simulated holograms covers the internal pixels (m=1:M−1, n=1:N−1), while the boundary pixels are assigned separately under the Neumann boundary condition to ensure the integrity of the divergence field.

The resulting reconstructed phase maps are evaluated against the analytical phase targets using RMSE, MAE, correlation coefficient, spatial error maps, and representative profiles. The phase-reconstruction results are presented in Fig 3, and the error distributions are presented in Figs 4–5.

**Fig 4:**
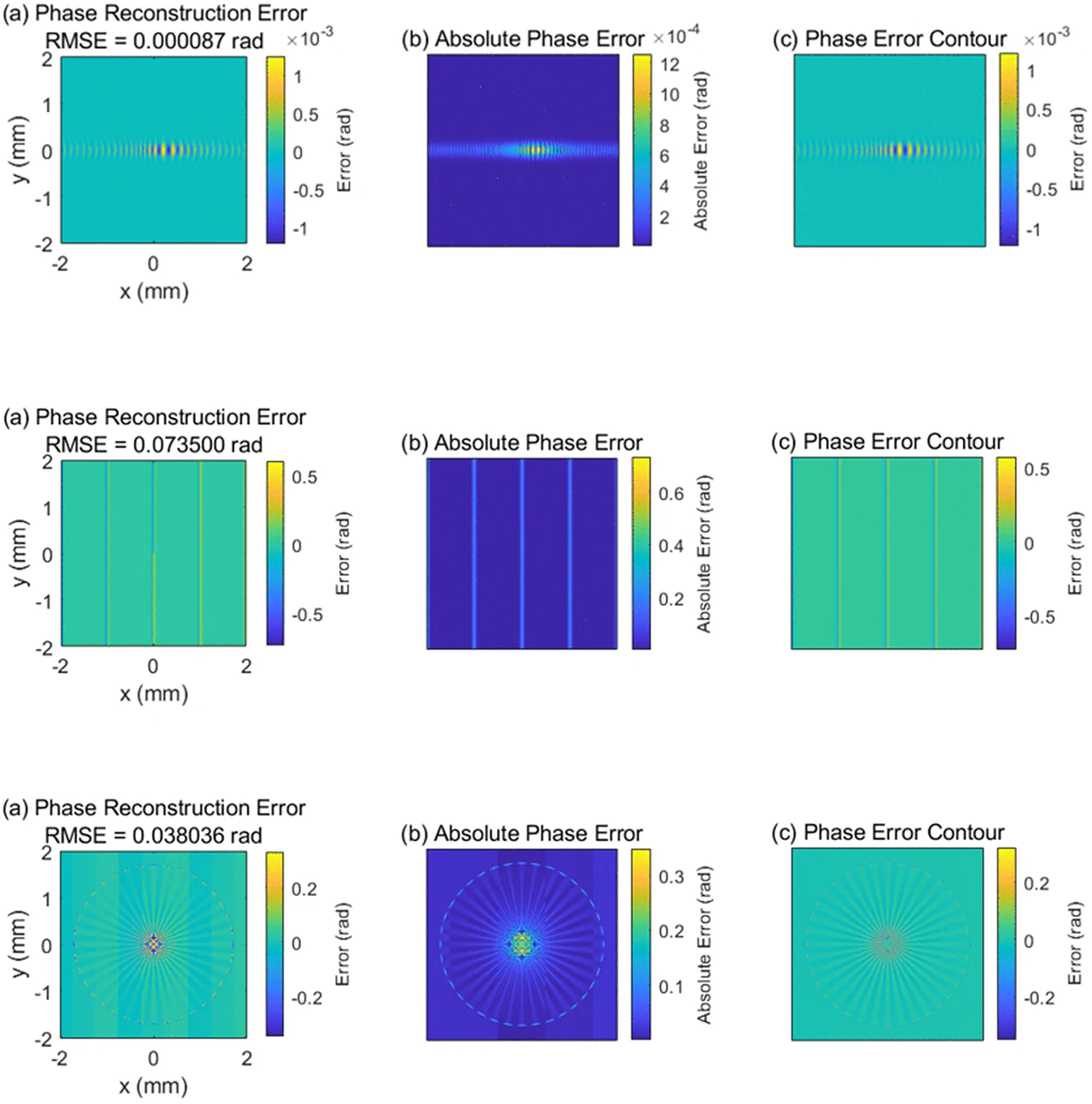
Spatial phase-reconstruction error analysis. The panels show the signed error map, absolute error map, and representative error statistics for the three benchmark targets. Spatial coordinates are reported in mm and error is reported in rad.

**Fig 5:**
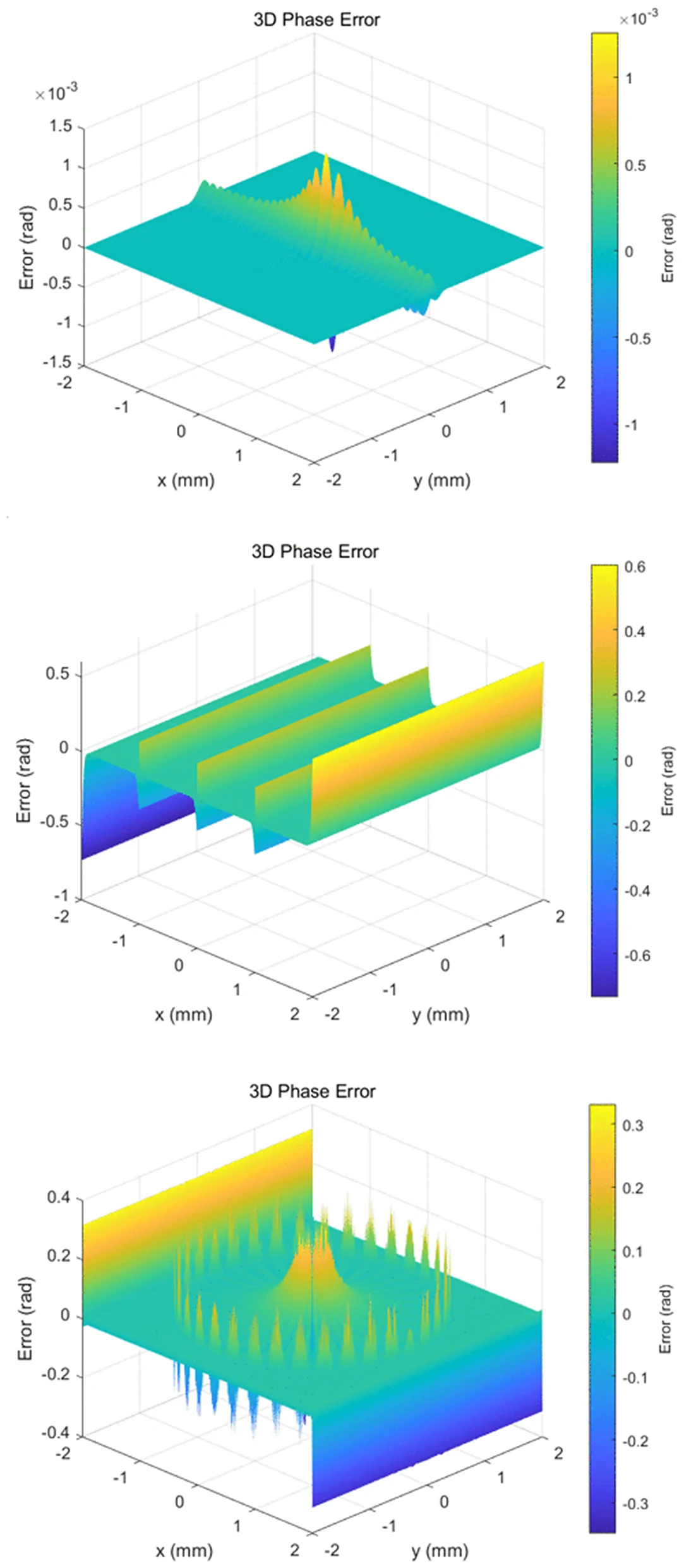
Three-dimensional error surface.

## Results and discussion

### Quality evaluation of simulated holograms

#### Analysis of fringe modulation effect.

The simulated hologram and fringe-modulation results for the three benchmark targets are summarized in Fig 1.

For the Gaussian target, the smoothly varying phase produces a correspondingly gradual modulation of the carrier fringe. The center-line comparison shows that the phase modulation changes the local fringe phase while preserving the underlying carrier structure.

The four-level step target produces abrupt fringe-phase changes at the prescribed step boundaries, as shown in Fig 1.

The discontinuities are localized to the phase-transition positions, while the approximately constant phase regions retain nearly periodic carrier fringes. This provides a direct visual check that the prescribed phase increments are encoded in the simulated hologram.

The Siemens Star produces a radially varying fringe modulation because its phase varies with angular position. The highest spatial-frequency structure occurs near the center, where adjacent spokes are most closely spaced. This behavior is consistent with the use of spoke-pattern targets for coherent-imaging resolution assessment [9]. Because the present target is numerical, the observation is interpreted as a structural benchmark rather than an experimental resolution measurement.

For the Siemens Star, the reconstruction metrics and profile comparisons are evaluated against the known simulated phase map. The resulting correlation coefficient is reported as a quantitative indicator of structural consistency rather than as evidence that the numerical model fully reproduces an experimental specimen.

The frequency-domain filtering results for the three cases are shown in Fig 2. The Gaussian spectrum is comparatively concentrated and is isolated with a rectangular window, whereas the step and Siemens Star spectra contain broader high-frequency structure and are processed with Gaussian soft windows. These differences provide a controlled comparison of how filter shape affects reconstruction of smooth and edge-rich phase distributions.

For the Gaussian case, the reconstructed phase follows the analytical profile closely. The four-level step reconstruction preserves the four prescribed phase regions but shows localized deviations at the phase boundaries. The Siemens Star reconstruction retains the radial phase structure, with larger deviations in the high-spatial-frequency central region. The three reconstructed phase maps are compared in Fig 3.

The error distribution of the four-level step target is concentrated around the discontinuity boundaries. The overall RMSE and correlation coefficient quantify the global agreement, while the spatial error map identifies the phase-transition regions as the dominant source of deviation.

The boundary-localized error is consistent with the finite bandwidth of the selected spectral window: abrupt spatial changes require broader frequency content than smooth regions. This interpretation is limited to the present numerical configuration and is not a measurement of an experimental optical transfer function.

For the Siemens Star, the error distribution follows the radial sector boundaries and becomes more pronounced toward the center, where the local spatial frequency is highest. This behavior is consistent with the intended role of the target as a structured high-frequency benchmark [9].

The error histogram provides a complementary global view of the reconstruction. The distribution is centered near zero for the tested cases, while the width of the distribution differs between the smooth and high-frequency targets.

The error maps show that the largest deviations are spatially organized rather than randomly distributed. In particular, the step boundaries and the Siemens Star spoke transitions account for a substantial fraction of the local error.

#### Comprehensive performance evaluation.

The comprehensive reconstruction analysis is summarized in Figs 6–8. For the Gaussian target, the original and reconstructed phase values cluster closely around the ideal one-to-one relationship, and the representative profiles show small deviations near the steepest part of the phase distribution.

**Fig 6:**
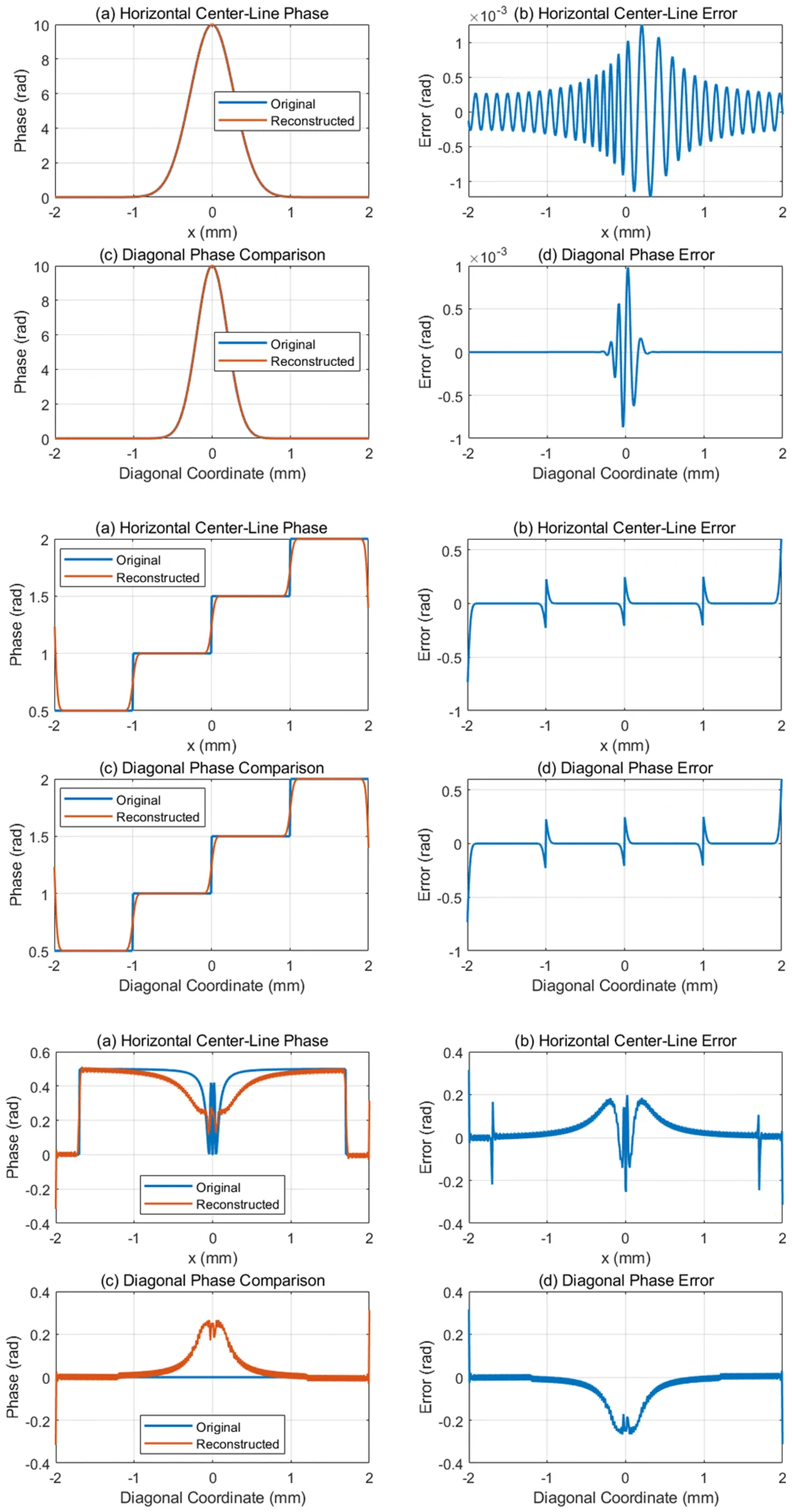
Representative profile analysis. The panels compare original and reconstructed phase profiles and corresponding errors along the horizontal center line and diagonal direction for the three benchmark targets. Profile coordinates are reported in mm; phase and error are reported in rad.

**Fig 7:**
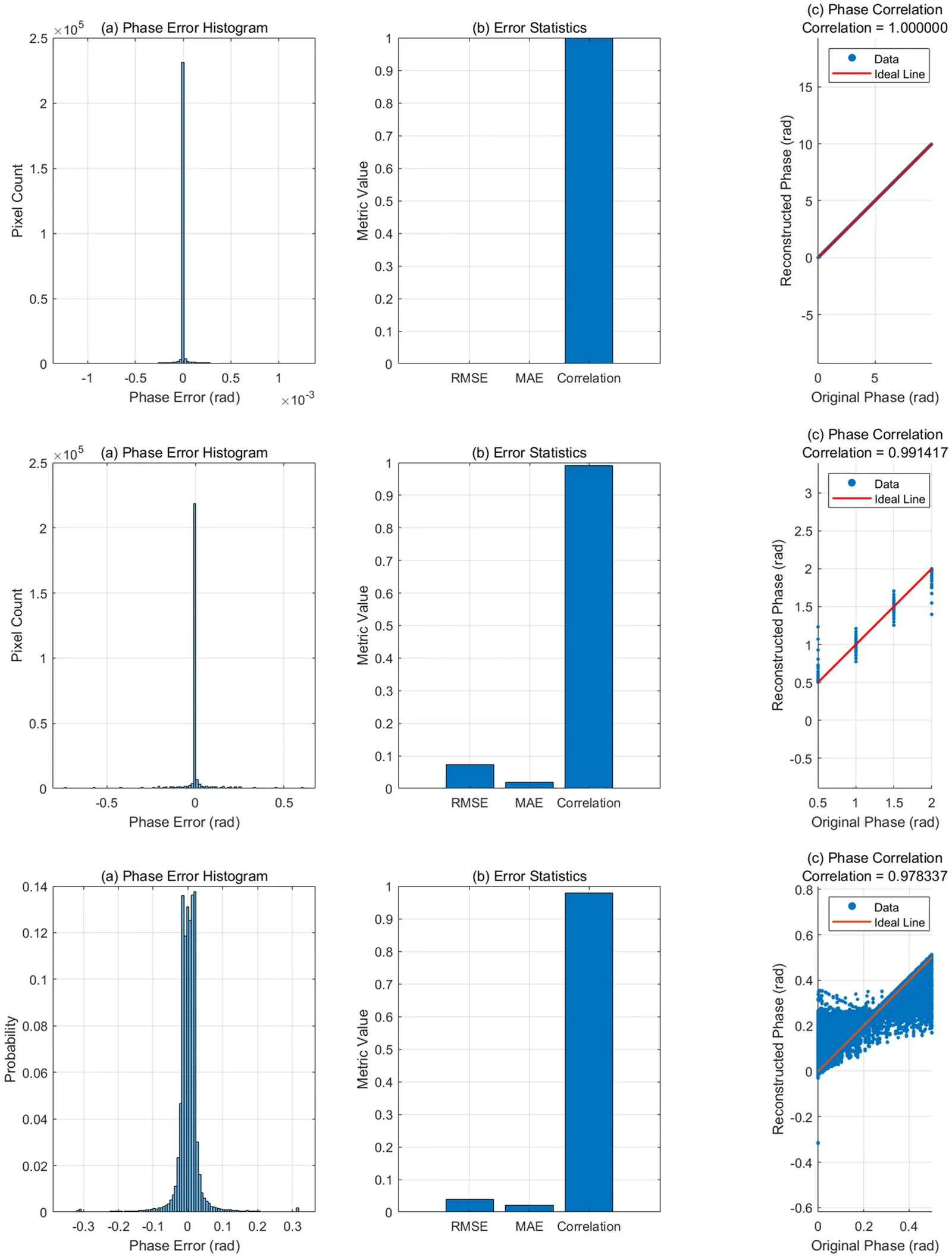
Statistical reconstruction evaluation. The panels show the phase-error histogram, RMSE/MAE/correlation summary, and original-versus-reconstructed phase scatter plot for the three benchmark targets. Phase and error are reported in rad; histogram values are reported as pixel counts or probability according to the displayed panel.

**Fig 8:**
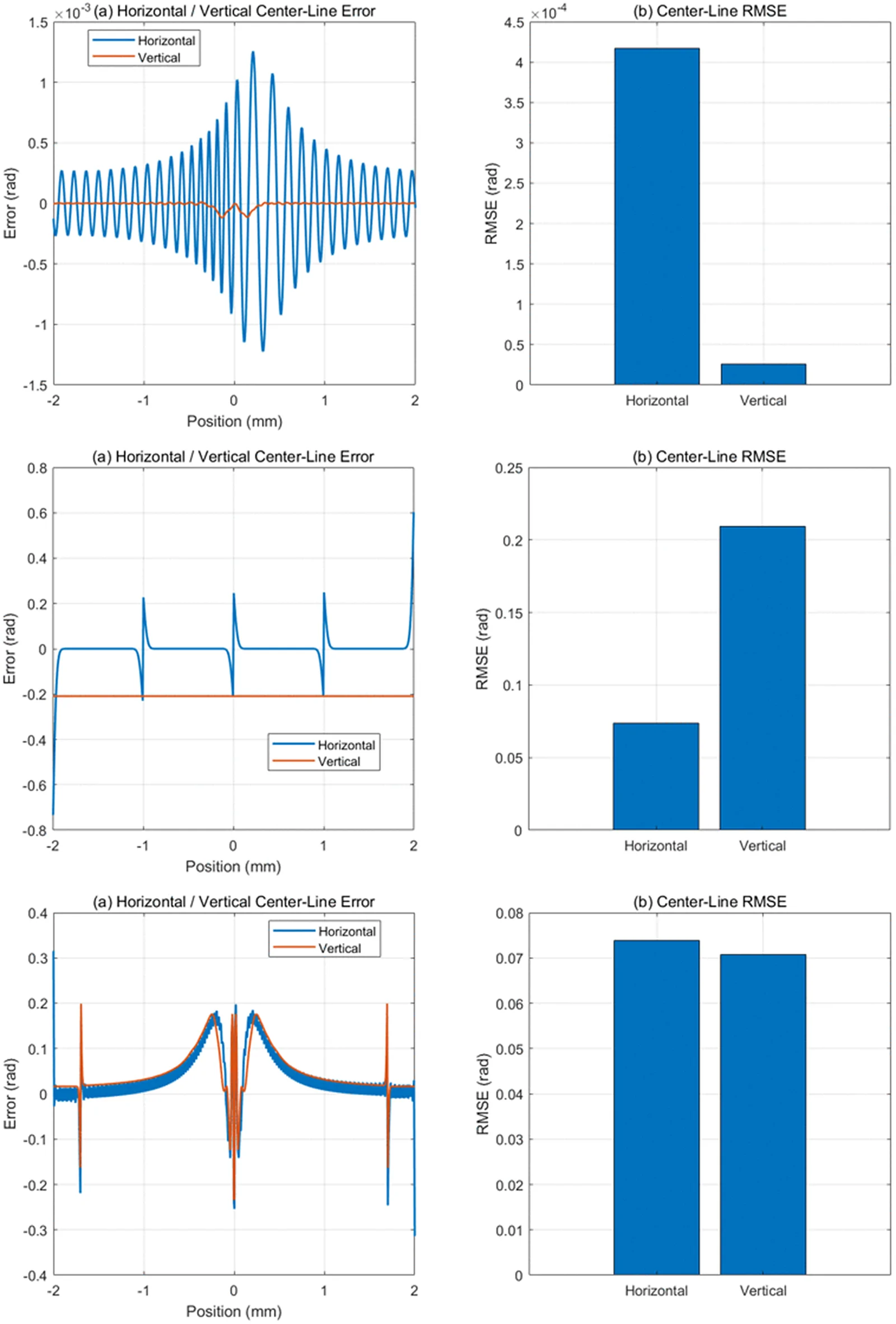
Horizontal and vertical center-line error analysis. The panels compare the two directional error profiles and the corresponding center-line RMSE values for the three benchmark targets. Position is reported in mm and error/RMSE is reported in rad.

The Gaussian diagonal profile reproduces the broad phase maximum and the reconstructed curve remains close to the analytical curve. The diagonal error remains small compared with the 10-rad phase range of the target.

The horizontal and vertical center-line error profiles provide directional information about the reconstruction. Their magnitudes remain substantially smaller than the phase dynamic range, with the largest deviations occurring near the region of maximum phase gradient.

For the four-level step target, the scatter plot forms four clusters corresponding to the prescribed phase levels, while the profile analysis shows that the largest deviations occur near the step boundaries.

The diagonal profile preserves the four-level structure and reveals localized edge deviations. This behavior is consistent with the sensitivity of abrupt phase transitions to finite-bandwidth spectral filtering.

The horizontal and vertical center-line errors are directionally different because the step boundaries are oriented along one image direction and the carrier is also oriented along the x direction. The observed asymmetry is therefore treated as a property of the simulated configuration rather than as a universal feature of step-phase holography.

For the Siemens Star, the scatter plot and profile comparisons show preservation of the dominant radial phase structure, with increased deviations near high-frequency spoke transitions. The results extend the benchmark from smooth and one-dimensional discontinuous structures to a spatially distributed high-frequency pattern.

The Siemens Star diagonal profile follows the main phase modulation of the analytical target, while deviations become more visible where the profile crosses closely spaced radial structures. The result is consistent with the spatial error map in Fig 5.

The horizontal and vertical center-line error profiles further show that the largest deviations occur around rapidly varying phase regions. These directional profiles are useful for identifying spatially localized reconstruction errors but should not be interpreted as a formal resolution measurement.

Taken together, the three benchmark cases demonstrate that the numerical pipeline can reproduce the main phase structure of smooth, discontinuous, and radially varying targets under the tested noise-free simulation conditions.

For the Gaussian model, the measured global RMSE is 0.000087 rad. This small error is consistent with the smooth phase profile and the controlled, noise-free simulation.

For the four-level step model, the global RMSE is 0.073500 rad and the correlation coefficient is 0.991417. The larger error relative to the Gaussian case is concentrated near the phase-transition boundaries.

For the Siemens Star model, the global RMSE is 0.038036 rad and the reconstruction retains the main radial structure. The larger local deviations occur in high-spatial-frequency transition regions.

The reported numerical results characterize this specific implementation and parameter set. They should not be interpreted as direct evidence of experimental real-time performance, optical resolution, or robustness to acquisition noise.

The dominant error mechanism observed in the simulations is therefore associated with rapid phase variation and finite spectral support. In future work, amplitude modulation, noise, speckle, aberration, and experimental detector effects should be introduced systematically, because these factors are known to influence practical holographic reconstruction [10, 13, 14].

#### Strengths and limitations.

The principal strength of this study is the explicit analytical ground truth, which permits reconstruction errors to be separated from experimental uncertainty. The use of three structurally different phase targets also provides a controlled comparison of smooth, discontinuous, and radially varying phase content. The principal limitations are that the simulations are noise-free, phase-only, and two-dimensional; they do not include amplitude modulation, speckle, optical aberration, detector nonuniformity, multiple scattering, or experimentally measured three-dimensional specimens. In addition, the three cases are benchmark examples rather than a statistically representative dataset, and therefore they should not be used to support claims about the prevalence of particular phase distributions in industrial or biomedical imaging. These limitations are important when interpreting the results and when considering the use of the simulations for later deep-learning studies [10–14].

## Conclusion

This study presents a controlled numerical framework for generating and reconstructing off-axis digital holograms with analytically known phase ground truth. Three benchmark phase structures—a smooth Gaussian distribution, a four-level step distribution, and a Siemens Star—were used to examine reconstruction behavior under different spatial-frequency characteristics. Object-specific frequency-domain filtering followed by DCT-based global phase unwrapping recovered the main phase structures in all three tested cases. The dominant errors occurred near abrupt or high-spatial-frequency phase transitions. The framework is therefore suitable as a reproducible baseline for algorithm verification and for subsequent comparison with more diverse experimental or learning-based datasets.

### Main contributions

1. A reproducible numerical benchmark with explicit phase ground truth is established for controlled evaluation of off-axis hologram reconstruction.
2. Object-specific frequency-domain windows are compared across smooth, discontinuous, and radially varying phase structures, revealing how spectral content relates to localized reconstruction error.
3. The study explicitly separates controlled numerical benchmarking from experimental validation and deep-learning dataset development, reducing the risk of overinterpreting the limited synthetic cases.

### Future work

1. Extend the simulations by introducing amplitude modulation, detector noise, speckle, optical aberration, illumination nonuniformity, and multiple depth planes, followed by validation against experimentally recorded holograms [10, 13, 14].
2. Develop larger and more diverse datasets before using the simulations for deep-learning-based phase reconstruction, with independent training, validation, and test sets and explicit reporting of dataset size and variation [11, 12].
3. Extend the numerical model toward experimentally validated three-dimensional objects and, where appropriate, multi-wavelength or color holography [15, 16].

## Supporting information

S1 Code Full MATLAB implementation code for the off-axis hologram numerical generation, object-specific frequency-domain filtering, and phase reconstruction procedures described in this work. The code is fully reproducible for all simulation results presented in the manuscript, compatible with MATLAB R2020a and later versions, and requires no additional third-party toolboxes.
(DOCX)
