# Supplementary material for: Controlled numerical generation and reconstruction of off-axis digital holograms with object-specific frequency-domain filtering
Source: PLoS One. 2026 Oct 22;21(10):e0360375. doi: 10.1371/journal.pone.0360375 (PMC13690232; doi:10.1371/journal.pone.0360375)
Supplement: Supplementary file 1 [file pone.0360375.s001.docx]

% Gaussian Model: Original Phase vs Reconstructed Phase

% Phase Error Analysis

% Unit Specification:

%	Spatial coordinate: mm

%	Intensity: a.u.

%	Phase: rad

%	Phase error: rad

%	Spectrum coordinate: pixel^-1

%% =========================================================

clear;

clc;

close all;

%% =========================================================

% 1. Parameter Settings

%% =========================================================

N = 512;

L0 = 4; % Calculation domain size (mm)

dx = L0 / N; % Pixel size (mm)

lambda = 632.8e-6; % Wavelength (mm)

phi0 = 10; % Peak value of Gaussian phase (rad)

sigma_pix = 50;

%% =========================================================

% 2. Coordinate Construction

%% =========================================================

x = linspace(-L0/2,L0/2,N);

y = linspace(-L0/2,L0/2,N);

[X,Y] = meshgrid(x,y);

%% =========================================================

% 3. Original Gaussian Phase Model

%% =========================================================

sigma_mm = sigma_pix * dx;

phi_true = phi0 .* exp( ...

-(X.^2 + Y.^2) / sigma_mm^2 );

%% =========================================================

% 4. Generate Reference Fringe

%% =========================================================

f = 40;

a0 = 0.5;

b0 = 0.5;

[Xp,~] = meshgrid(1:N,1:N);

I_ref = a0 + ...

b0*cos(2*pi*f*Xp/N);

%% =========================================================

% 5. Generate Hologram

%% =========================================================

I_holo = a0 + ...

b0*cos(2*pi*f*Xp/N + phi_true);

%% =========================================================

% 6. Center Row Data

%% =========================================================

center_row = round(N/2);

ref_center = I_ref(center_row,:);

holo_center = I_holo(center_row,:);

fringe_diff = holo_center - ref_center;

fringe_2D_diff = I_holo - I_ref;

%% =========================================================

% 7. Fringe Difference Statistics

%% =========================================================

fprintf('\n');

fprintf('=========================================\n');

fprintf('Gaussian Model Fringe Difference Analysis\n');

fprintf('=========================================\n');

fprintf('Maximum fringe difference in center row = %.8f\n', ...

max(abs(fringe_diff)));

fprintf('Maximum 2D fringe difference	= %.8f\n', ...

max(abs(fringe_2D_diff(:))));

fprintf('Mean 2D fringe difference	= %.8f\n', ...

mean(abs(fringe_2D_diff(:))));

fprintf('=========================================\n');

%% =========================================================

% Figure 1: Fringe Generation and Difference Analysis

%

% (a) Reference Fringe

% (b) Simulated Hologram

% (c) 2D Fringe Difference

% (d) Center-Line Fringe Difference

% (e) Center-Line Fringe Comparison

% (f) Center-Region Enlargement

%% =========================================================

figure('Color','w');

%% ---------- (a) Reference Fringe ----------

subplot(2,3,1);

imagesc(x,y,I_ref);

axis image;

set(gca,'YDir','normal');

xlabel('x (mm)');

ylabel('y (mm)');

title('(a) Reference Fringe');

cb = colorbar;

ylabel(cb,'Intensity (a.u.)');

%% ---------- (b) Simulated Hologram ----------

subplot(2,3,2);

imagesc(x,y,I_holo);

axis image;

set(gca,'YDir','normal');

xlabel('x (mm)');

ylabel('y (mm)');

title('(b) Simulated Hologram');

cb = colorbar;

ylabel(cb,'Intensity (a.u.)');

%% ---------- (c) 2D Fringe Difference ----------

subplot(2,3,3);

imagesc(x,y,fringe_2D_diff);

axis image;

set(gca,'YDir','normal');

xlabel('x (mm)');

ylabel('y (mm)');

title('(c) 2D Fringe Difference');

cb = colorbar;

ylabel(cb,'\Delta I (a.u.)');

%% ---------- (d) Center-Line Fringe Difference ----------

subplot(2,3,4);

plot(x,fringe_diff,...

'LineWidth',1.3);

grid on;

xlabel('x (mm)');

ylabel('\Delta I (a.u.)');

title('(d) Center-Line Fringe Difference');

%% ---------- (e) Center-Line Fringe Comparison ----------

subplot(2,3,5);

plot(x,ref_center,...

'LineWidth',1.5);

hold on;

plot(x,holo_center,...

'LineWidth',1.2);

grid on;

xlabel('x (mm)');

ylabel('Intensity (a.u.)');

title('(e) Center-Line Fringe Comparison');

legend('Reference','Hologram',...

'Location','best');

%% ---------- (f) Center-Region Enlargement ----------

subplot(2,3,6);

plot(x,fringe_diff,...

'LineWidth',1.3);

xlim([-0.6 0.6]);

grid on;

xlabel('x (mm)');

ylabel('\Delta I (a.u.)');

title('(f) Center-Region Enlargement');

%% =========================================================

% 8. FFT Spectrum

%% =========================================================

F = fft2(I_holo);

F_shift = fftshift(F);

Spectrum = log(1 + abs(F_shift));

Spectrum = Spectrum ./ max(Spectrum(:));

%% =========================================================

% 9. +1 Order Spectrum Position

%% =========================================================

peakX = 308;

peakY = 256;

%% =========================================================

% 10. Construct Rectangular Filter Window

%

% Half width = 40 pixel

% Half height = 40 pixel

%% =========================================================

dx_win = 40;

dy_win = 40;

filter = zeros(N);

x1 = peakX - dx_win;

x2 = peakX + dx_win;

y1 = peakY - dy_win;

y2 = peakY + dy_win;

filter(y1:y2,x1:x2) = 1;

%% =========================================================

% 11. Frequency Domain Filtering

%% =========================================================

F_filter = F_shift .* filter;

Spec_filter = log(1 + abs(F_filter));

Spec_filter = ...

Spec_filter ./ max(Spec_filter(:));

%% =========================================================

% Figure 2: Spectrum and Rectangular Frequency Filtering

%

% (a) Hologram Spectrum

% (b) Rectangular Filter Window

% (c) Filtered Spectrum

%% =========================================================

figure('Color','w');

%% ---------- (a) Hologram Spectrum ----------

subplot(1,3,1);

imagesc( ...

(-N/2:N/2-1),...

(-N/2:N/2-1),...

Spectrum);

axis image;

set(gca,'YDir','normal');

xlabel('f_x (pixel^{-1})');

ylabel('f_y (pixel^{-1})');

title('(a) Hologram Spectrum');

cb = colorbar;

ylabel(cb,'Normalized Magnitude');

%% ---------- (b) Rectangular Window ----------

subplot(1,3,2);

imagesc( ...

(-N/2:N/2-1),...

(-N/2:N/2-1),...

filter);

axis image;

set(gca,'YDir','normal');

xlabel('f_x (pixel^{-1})');

ylabel('f_y (pixel^{-1})');

title('(b) Rectangular Frequency Window');

cb = colorbar;

ylabel(cb,'Transmission');

%% ---------- (c) Filtered Spectrum ----------

subplot(1,3,3);

imagesc( ...

(-N/2:N/2-1),...

(-N/2:N/2-1),...

Spec_filter);

axis image;

set(gca,'YDir','normal');

xlabel('f_x (pixel^{-1})');

ylabel('f_y (pixel^{-1})');

title('(c) Filtered Spectrum');

cb = colorbar;

ylabel(cb,'Normalized Magnitude');

%% =========================================================

% 12. Inverse Fourier Reconstruction

%% =========================================================

F_inverse = ifftshift(F_filter);

U1 = ifft2(F_inverse);

%% =========================================================

% 13. Construct Reference Complex Field

%% =========================================================

U_ref = exp(1j*2*pi*f*Xp/N);

%% =========================================================

% 14. Complex Conjugate Multiplication

%% =========================================================

U_phase = U1 .* conj(U_ref);

%% =========================================================

% 15. Extract Wrapped Phase

%% =========================================================

phi_wrapped = angle(U_phase);

%% =========================================================

% 16. DCT Global Phase Unwrapping

%% =========================================================

%% ---------- Phase Gradient ----------

gx = diff(phi_wrapped,1,2);

gy = diff(phi_wrapped,1,1);

%% ---------- 2pi Normalization ----------

gx = atan2(sin(gx),cos(gx));

gy = atan2(sin(gy),cos(gy));

%% =========================================================

% 17. Construct Divergence Field

%% =========================================================

rho = zeros(N);

%% ---------- x direction ----------

rho(:,2:end-1) = ...

rho(:,2:end-1) + ...

gx(:,2:end) - gx(:,1:end-1);

%% ---------- y direction ----------

rho(2:end-1,:) = ...

rho(2:end-1,:) + ...

gy(2:end,:) - gy(1:end-1,:);

%% =========================================================

% 18. Neumann Boundary Condition

%% =========================================================

rho(:,1) = gx(:,1);

rho(:,end) = -gx(:,end);

rho(1,:) = rho(1,:) + gy(1,:);

rho(end,:) = rho(end,:) - gy(end,:);

%% =========================================================

% 19. DCT Solve Poisson Equation

%% =========================================================

rho_dct = dct2(rho);

[m,n] = meshgrid(0:N-1,0:N-1);

denominator = ...

2*cos(pi*m/N) + ...

2*cos(pi*n/N) - 4;

denominator(1,1) = 1;

phi_dct = rho_dct ./ denominator;

phi_dct(1,1) = 0;

%% =========================================================

% 20. Inverse DCT Recover Continuous Phase

%% =========================================================

phi_recon = idct2(phi_dct);

%% =========================================================

% 21. Remove Global Offset

%% =========================================================

offset = ...

mean(phi_true(:)-phi_recon(:));

phi_recon = phi_recon + offset;

%% =========================================================

% 22. Phase Error

%% =========================================================

error_map = phi_true - phi_recon;

abs_error = abs(error_map);

%% =========================================================

% 23. RMSE Calculation

%% =========================================================

rmse = sqrt(mean(error_map(:).^2));

%% =========================================================

% 24. MAE Calculation

%% =========================================================

mae = mean(abs(error_map(:)));

%% =========================================================

% 25. Correlation Coefficient

%% =========================================================

R = corrcoef( ...

phi_true(:),...

phi_recon(:));

corr_value = R(1,2);

%% =========================================================

% Figure 3: Phase Reconstruction Results

%

% (a) Wrapped Phase

% (b) Original Gaussian Phase

% (c) Reconstructed Phase

%% =========================================================

figure('Color','w');

%% ---------- (a) Wrapped Phase ----------

subplot(1,3,1);

imagesc(x,y,phi_wrapped);

axis image;

set(gca,'YDir','normal');

xlabel('x (mm)');

ylabel('y (mm)');

title('(a) Wrapped Phase');

cb = colorbar;

ylabel(cb,'Phase (rad)');

%% ---------- (b) Original Gaussian Phase ----------

subplot(1,3,2);

surf(X,Y,phi_true,...

'EdgeColor','none');

shading interp;

view(45,30);

xlabel('x (mm)');

ylabel('y (mm)');

zlabel('Phase (rad)');

title('(b) Original Gaussian Phase');

cb = colorbar;

ylabel(cb,'Phase (rad)');

%% ---------- (c) Reconstructed Phase ----------

subplot(1,3,3);

surf(X,Y,phi_recon,...

'EdgeColor','none');

shading interp;

view(45,30);

xlabel('x (mm)');

ylabel('y (mm)');

zlabel('Phase (rad)');

title('(c) Reconstructed Phase');

cb = colorbar;

ylabel(cb,'Phase (rad)');

%% =========================================================

% Figure 4: 2D Phase Error Analysis

%

% (a) Phase Error Map

% (b) Absolute Phase Error

% (c) Phase Error Contour

%3D Phase Error Surface

%% =========================================================

figure('Color','w');

%% ---------- (a) Phase Error Map ----------

subplot(1,3,1);

imagesc(x,y,error_map);

axis image;

set(gca,'YDir','normal');

xlabel('x (mm)');

ylabel('y (mm)');

title({ ...

'(a) Phase Reconstruction Error', ...

['RMSE = ',...

num2str(rmse,'%.6f'),...

' rad']});

cb = colorbar;

ylabel(cb,'Error (rad)');

%% ---------- (b) Absolute Phase Error ----------

subplot(1,3,2);

imagesc(x,y,abs_error);

axis image;

set(gca,'YDir','normal');

xlabel('x (mm)');

ylabel('y (mm)');

title('(b) Absolute Phase Error');

cb = colorbar;

ylabel(cb,'Absolute Error (rad)');

%% ---------- (c) Error Contour ----------

subplot(1,3,3);

contourf(X,Y,error_map,50,...

'LineColor','none');

axis image;

xlabel('x (mm)');

ylabel('y (mm)');

title('(c) Phase Error Contour');

cb = colorbar;

ylabel(cb,'Error (rad)');

%% ---------- 3D Error Surface ----------

figure;

surf(X,Y,error_map,...

'EdgeColor','none');

shading interp;

view(45,30);

xlabel('x (mm)');

ylabel('y (mm)');

zlabel('Error (rad)');

title(' 3D Phase Error');

cb = colorbar;

ylabel(cb,'Error (rad)');

set(gcf,'color','w');

set(gca,'color','w');

%% =========================================================

% 26. Center Profile

%% =========================================================

center_true = ...

phi_true(center_row,:);

center_recon = ...

phi_recon(center_row,:);

center_error = ...

error_map(center_row,:);

%% =========================================================

% 27. Diagonal Profile

%% =========================================================

diag_true = diag(phi_true);

diag_recon = diag(phi_recon);

diag_error = ...

diag_true - diag_recon;

diag_coord = ...

linspace(-L0/2,L0/2,N);

%% =========================================================

% Figure 5: Typical Profile Analysis

%

% (a) Horizontal Center-Line Phase

% (b) Horizontal Center-Line Error

% (c) Diagonal Phase Comparison

% (d) Diagonal Phase Error

%% =========================================================

figure('Color','w');

%% ---------- (a) Center-Line Phase ----------

subplot(2,2,1);

plot(x,center_true,...

'LineWidth',1.5);

hold on;

plot(x,center_recon,...

'LineWidth',1.2);

grid on;

xlabel('x (mm)');

ylabel('Phase (rad)');

title('(a) Horizontal Center-Line Phase');

legend('Original','Reconstructed',...

'Location','best');

%% ---------- (b) Center-Line Error ----------

subplot(2,2,2);

plot(x,center_error,...

'LineWidth',1.3);

grid on;

xlabel('x (mm)');

ylabel('Error (rad)');

title('(b) Horizontal Center-Line Error');

%% ---------- (c) Diagonal Phase Comparison ----------

subplot(2,2,3);

plot(diag_coord,diag_true,...

'LineWidth',1.5);

hold on;

plot(diag_coord,diag_recon,...

'LineWidth',1.2);

grid on;

xlabel('Diagonal Coordinate (mm)');

ylabel('Phase (rad)');

title('(c) Diagonal Phase Comparison');

legend('Original','Reconstructed',...

'Location','best');

%% ---------- (d) Diagonal Phase Error ----------

subplot(2,2,4);

plot(diag_coord,diag_error,...

'LineWidth',1.3);

grid on;

xlabel('Diagonal Coordinate (mm)');

ylabel('Error (rad)');

title('(d) Diagonal Phase Error');

%% =========================================================

% Figure 6: Error Statistics and Correlation

%

% (a) Error Histogram

% (b) RMSE / MAE / Correlation

% (c) Original vs Reconstructed Phase Scatter Plot

%% =========================================================

figure('Color','w');

%% ---------- (a) Error Histogram ----------

subplot(1,3,1);

histogram(error_map(:),100);

grid on;

xlabel('Phase Error (rad)');

ylabel('Pixel Count');

title('(a) Phase Error Histogram');

%% ---------- (b) Statistical Metrics ----------

subplot(1,3,2);

bar([rmse mae corr_value]);

grid on;

set(gca,...

'XTick',1:3,...

'XTickLabel',...

{'RMSE','MAE','Correlation'});

ylabel('Metric Value');

title('(b) Error Statistics');

%% ---------- (c) Phase Correlation Scatter Plot ----------

subplot(1,3,3);

scatter( ...

phi_true(:),...

phi_recon(:),...

8,...

'filled');

hold on;

minVal = min(phi_true(:));

maxVal = max(phi_true(:));

plot( ...

[minVal maxVal],...

[minVal maxVal],...

'r',...

'LineWidth',1.5);

grid on;

axis equal;

xlabel('Original Phase (rad)');

ylabel('Reconstructed Phase (rad)');

title({ ...

'(c) Phase Correlation', ...

['Correlation = ',...

num2str(corr_value,'%.6f')]});

legend('Data','Ideal Line',...

'Location','northwest');

%% =========================================================

% 28. Horizontal / Vertical Center-Line Error

%% =========================================================

center_col = round(N/2);

horizontal_error = ...

error_map(center_row,:);

vertical_error = ...

error_map(:,center_col);

%% =========================================================

% 29. Center-Line RMSE

%% =========================================================

horizontal_rmse = ...

sqrt(mean(horizontal_error.^2));

vertical_rmse = ...

sqrt(mean(vertical_error.^2));

%% =========================================================

% Figure 7: Horizontal / Vertical Center-Line Error

%

% (a) Horizontal / Vertical Center-Line Error Comparison

% (b) Center-Line RMSE

%% =========================================================

figure('Color','w');

%% ---------- (a) Center-Line Error Comparison ----------

subplot(1,2,1);

plot(x,horizontal_error,...

'LineWidth',1.5);

hold on;

plot(y,vertical_error,...

'LineWidth',1.3);

grid on;

xlabel('Position (mm)');

ylabel('Error (rad)');

title('(a) Horizontal / Vertical Center-Line Error');

legend('Horizontal','Vertical',...

'Location','best');

%% ---------- (b) Center-Line RMSE ----------

subplot(1,2,2);

bar([ ...

horizontal_rmse,...

vertical_rmse]);

grid on;

set(gca,...

'XTick',1:2,...

'XTickLabel',...

{'Horizontal','Vertical'});

ylabel('RMSE (rad)');

title('(b) Center-Line RMSE');

%% =========================================================

% 30. Final Result Output

%% =========================================================

fprintf('\n');

fprintf('=========================================\n');

fprintf('Gaussian Phase Reconstruction Error Analysis Completed\n');

fprintf('=========================================\n');

fprintf('RMSE	= %.8f rad\n',...

rmse);

fprintf('MAE	= %.8f rad\n',...

mae);

fprintf('Correlation Coefficient = %.8f\n',...

corr_value);

fprintf('\n');

fprintf('Maximum Absolute Error	= %.8f rad\n',...

max(abs(error_map(:))));

fprintf('Minimum Absolute Error	= %.8f rad\n',...

min(abs(error_map(:))));

fprintf('\n');

fprintf('Maximum Diagonal Error	= %.8f rad\n',...

max(abs(diag_error)));

fprintf('Maximum Center-Line Error = %.8f rad\n',...

max(abs(center_error)));

fprintf('\n');

fprintf('Horizontal Center-Line RMSE = %.8f rad\n',...

horizontal_rmse);

fprintf('Vertical Center-Line RMSE	= %.8f rad\n',...

vertical_rmse);

fprintf('\n');

fprintf('=========================================\n');

fprintf('Unified Figure Specification:\n');

fprintf('Spatial Coordinate	= mm\n');

fprintf('Intensity	= a.u.\n');

fprintf('Phase	= rad\n');

fprintf('Phase Error	= rad\n');

fprintf('Spectrum Coordinate	= pixel^{-1}\n');

fprintf('=========================================\n');

% Four-Level Step Phase Model Digital Holography Simulation

% Four phase levels:

% 0.5 rad

% 1.0 rad

% 1.5 rad

% 2.0 rad

% Complete workflow:

% Unified plot specification:

%	Spatial coordinate: mm

%	Intensity: a.u.

%	Phase: rad

%	Phase error: rad

%	Frequency: pixel^-1

%

%% =========================================================

clear;

clc;

close all;

%% =========================================================

% 1. Parameter Settings

%% =========================================================

N = 512;

L0 = 4; % mm

dx = L0 / N; % mm/pixel

lambda = 632.8e-6; % mm

z = 100; % mm

l = 300; % mm

k = 100;

%% =========================================================

% Dense fringe configuration for step object

%% =========================================================

p = 4;

f = 1/p; % pixel^-1

%% =========================================================

% 2. Coordinate Construction

%% =========================================================

[X,Y] = meshgrid(1:N,1:N);

%% ---------- Actual spatial coordinate ----------

x = ((1:N) - (N+1)/2) * dx;

y = ((1:N) - (N+1)/2) * dx;

[Xmm,Ymm] = meshgrid(x,y);

%% =========================================================

% 3. Construct Four-Level Step Phase Model

%% =========================================================

phi_true = zeros(N);

%% ---------- Level 1 ----------

phi_true(:,1:128) = 0.5;

%% ---------- Level 2 ----------

phi_true(:,129:256) = 1.0;

%% ---------- Level 3 ----------

phi_true(:,257:384) = 1.5;

%% ---------- Level 4 ----------

phi_true(:,385:512) = 2.0;

%% =========================================================

% 4. Generate Reference Fringe

%% =========================================================

a0 = 0.5;

b0 = 0.5;

I_ref = a0 + ...

b0*cos(2*pi*f*X);

%% =========================================================

% 5. Generate Step-Modulated Hologram

%% =========================================================

I_holo = a0 + ...

b0*cos(2*pi*f*X + phi_true);

%% =========================================================

% 6. Center Row Data

%% =========================================================

center_row = round(N/2);

ref_center = I_ref(center_row,:);

holo_center = I_holo(center_row,:);

fringe_diff = holo_center - ref_center;

%% =========================================================

% 7. 2D Fringe Difference

%% =========================================================

fringe_2D_diff = I_holo - I_ref;

%% =========================================================

% 8. Output Fringe Difference Statistics

%% =========================================================

fprintf('\n');

fprintf('=========================================\n');

fprintf('Fringe Difference Analysis Completed\n');

fprintf('=========================================\n');

fprintf('Maximum fringe difference in center row = %.8f\n', ...

max(abs(fringe_diff)));

fprintf('Maximum 2D fringe difference	= %.8f\n', ...

max(abs(fringe_2D_diff(:))));

fprintf('Mean 2D fringe difference	= %.8f\n', ...

mean(abs(fringe_2D_diff(:))));

fprintf('=========================================\n');

%% =========================================================

% Figure 1: Fringe Generation and Difference Analysis

% (Positions fully aligned with Gaussian model Figure 1)

%

% (a) Reference Fringe

% (b) Simulated Hologram

% (c) 2D Fringe Difference

% (d) Center-Line Fringe Difference

% (e) Center-Line Fringe Comparison

% (f) Center-Region Enlargement

%% =========================================================

figure('Color','w');

%% ---------- (a) Reference Fringe ----------

subplot(2,3,1);

imagesc(x,y,I_ref);

axis image;

set(gca,'YDir','normal');

xlabel('x (mm)');

ylabel('y (mm)');

title('(a) Reference Fringe');

cb = colorbar;

ylabel(cb,'Intensity (a.u.)');

%% ---------- (b) Simulated Hologram ----------

subplot(2,3,2);

imagesc(x,y,I_holo);

axis image;

set(gca,'YDir','normal');

xlabel('x (mm)');

ylabel('y (mm)');

title('(b) Simulated Hologram');

cb = colorbar;

ylabel(cb,'Intensity (a.u.)');

%% ---------- (c) 2D Fringe Difference ----------

subplot(2,3,3);

imagesc(x,y,fringe_2D_diff);

axis image;

set(gca,'YDir','normal');

xlabel('x (mm)');

ylabel('y (mm)');

title('(c) 2D Fringe Difference');

cb = colorbar;

ylabel(cb,'\Delta I (a.u.)');

%% ---------- (d) Center-Line Fringe Difference ----------

subplot(2,3,4);

plot(x,fringe_diff,'LineWidth',1.3);

grid on;

xlabel('x (mm)');

ylabel('\Delta I (a.u.)');

title('(d) Center-Line Fringe Difference');

%% ---------- (e) Center-Line Fringe Comparison ----------

subplot(2,3,5);

plot(x,ref_center,'LineWidth',1.5);

hold on;

plot(x,holo_center,'LineWidth',1.2);

grid on;

xlabel('x (mm)');

ylabel('Intensity (a.u.)');

title('(e) Center-Line Fringe Comparison');

legend('Reference','Hologram','Location','best');

%% ---------- (f) Center-Region Enlargement ----------

subplot(2,3,6);

plot(x,fringe_diff,'LineWidth',1.3);

xlim([-0.6 0.6]);

grid on;

xlabel('x (mm)');

ylabel('\Delta I (a.u.)');

title('(f) Center-Region Enlargement');

%% =========================================================

% 9. FFT Spectrum

%% =========================================================

F = fft2(I_holo);

F_shift = fftshift(F);

Spectrum = log(1 + abs(F_shift));

Spectrum = Spectrum ./ max(Spectrum(:));

%% =========================================================

% 10. Fixed +1 Order Spectrum Extraction

%% =========================================================

peakX = 384;

peakY = 256;

%% =========================================================

% 11. Dynamic Gaussian Window Soft Filtering

%% =========================================================

sigma_x = 18;

sigma_y = 18;

%% =========================================================

% Construct 2D Gaussian Filter Window

%% =========================================================

[u,v] = meshgrid(1:N,1:N);

filter = exp( ...

-((u-peakX).^2)/(2*sigma_x^2) ...

-((v-peakY).^2)/(2*sigma_y^2) );

%% =========================================================

% Normalization

%% =========================================================

filter = filter ./ max(filter(:));

%% =========================================================

% 12. Frequency Domain Filtering

%% =========================================================

F_filter = F_shift .* filter;

Spec_filter = log(1 + abs(F_filter));

Spec_filter = ...

Spec_filter ./ max(Spec_filter(:));

%% =========================================================

% Spectrum Coordinate

%

% Frequency coordinate after fftshift:

% Unit: pixel^-1

%% =========================================================

freq = (-N/2:N/2-1) / N;

%% =========================================================

% Figure 2: Spectrum and Gaussian Filtering

% (Positions fully aligned with Gaussian model Figure 2)

%

% (a) Hologram Spectrum

% (b) Gaussian Frequency Window

% (c) Filtered Spectrum

%% =========================================================

figure('Color','w');

%% ---------- (a) Hologram Spectrum ----------

subplot(1,3,1);

imagesc(freq,freq,Spectrum);

axis image;

set(gca,'YDir','normal');

xlabel('f_x (pixel^{-1})');

ylabel('f_y (pixel^{-1})');

title('(a) Hologram Spectrum');

cb = colorbar;

ylabel(cb,'Normalized Magnitude');

%% ---------- (b) Gaussian Window ----------

subplot(1,3,2);

imagesc(freq,freq,filter);

axis image;

set(gca,'YDir','normal');

xlabel('f_x (pixel^{-1})');

ylabel('f_y (pixel^{-1})');

title('(b) Gaussian Frequency Window');

cb = colorbar;

ylabel(cb,'Transmission');

%% ---------- (c) Filtered Spectrum ----------

subplot(1,3,3);

imagesc(freq,freq,Spec_filter);

axis image;

set(gca,'YDir','normal');

xlabel('f_x (pixel^{-1})');

ylabel('f_y (pixel^{-1})');

title('(c) Filtered Spectrum');

cb = colorbar;

ylabel(cb,'Normalized Magnitude');

%% =========================================================

% 13. Inverse Fourier Reconstruction

%% =========================================================

F_inverse = ifftshift(F_filter);

U1 = ifft2(F_inverse);

%% =========================================================

% 14. Construct Reference Complex Field

%% =========================================================

U_ref = exp(1j*2*pi*f*X);

%% =========================================================

% 15. Complex Conjugate Multiplication

%% =========================================================

U_phase = U1 .* conj(U_ref);

%% =========================================================

% 16. Extract Wrapped Phase

%% =========================================================

phi_wrapped = angle(U_phase);

%% =========================================================

% 17. DCT Global Phase Unwrapping

%% =========================================================

gx = diff(phi_wrapped,1,2);

gy = diff(phi_wrapped,1,1);

%% ---------- 2pi Normalization ----------

gx = atan2(sin(gx),cos(gx));

gy = atan2(sin(gy),cos(gy));

%% =========================================================

% 18. Construct Divergence Field

%% =========================================================

rho = zeros(N);

%% ---------- x direction ----------

rho(:,2:end-1)= ...

rho(:,2:end-1)+ ...

gx(:,2:end)-gx(:,1:end-1);

%% ---------- y direction ----------

rho(2:end-1,:)= ...

rho(2:end-1,:)+ ...

gy(2:end,:)-gy(1:end-1,:);

%% =========================================================

% Neumann Boundary Condition

%% =========================================================

rho(:,1)=gx(:,1);

rho(:,end)=-gx(:,end);

rho(1,:)=rho(1,:)+gy(1,:);

rho(end,:)=rho(end,:)-gy(end,:);

%% =========================================================

% 19. DCT Solve Poisson Equation

%% =========================================================

rho_dct = dct2(rho);

[m,n] = meshgrid(0:N-1,0:N-1);

denominator = ...

2*cos(pi*m/N) + ...

2*cos(pi*n/N) - 4;

denominator(1,1)=1;

phi_dct = rho_dct ./ denominator;

phi_dct(1,1)=0;

%% =========================================================

% 20. Inverse DCT Recover Continuous Phase

%% =========================================================

phi_recon = idct2(phi_dct);

%% =========================================================

% 21. Remove Global Offset

%% =========================================================

offset = mean(phi_true(:)-phi_recon(:));

phi_recon = phi_recon + offset;

%% =========================================================

% 22. Phase Error Calculation

%% =========================================================

error_map = phi_true - phi_recon;

abs_error = abs(error_map);

%% =========================================================

% 23. RMSE Calculation

%% =========================================================

rmse = sqrt(mean(error_map(:).^2));

%% =========================================================

% 24. MAE Calculation

%% =========================================================

mae = mean(abs(error_map(:)));

%% =========================================================

% 25. Correlation Coefficient

%% =========================================================

R = corrcoef(phi_true(:),phi_recon(:));

corr_value = R(1,2);

%% =========================================================

% Figure 3: Phase Reconstruction Results

% (Positions fully aligned with Gaussian model Figure 3)

%

% (a) Wrapped Phase

% (b) Original Four-Level Step Phase

% (c) Reconstructed Step Phase

%% =========================================================

figure('Color','w');

%% ---------- (a) Wrapped Phase ----------

subplot(1,3,1);

imagesc(x,y,phi_wrapped);

axis image;

set(gca,'YDir','normal');

xlabel('x (mm)');

ylabel('y (mm)');

title('(a) Wrapped Phase');

cb = colorbar;

ylabel(cb,'Phase (rad)');

%% ---------- (b) Original Four-Level Phase ----------

subplot(1,3,2);

surf(Xmm,Ymm,phi_true,'EdgeColor','none');

shading interp;

view(45,30);

xlabel('x (mm)');

ylabel('y (mm)');

zlabel('Phase (rad)');

title('(b) Original Four-Level Phase');

cb = colorbar;

ylabel(cb,'Phase (rad)');

%% ---------- (c) Reconstructed Phase ----------

subplot(1,3,3);

surf(Xmm,Ymm,phi_recon,'EdgeColor','none');

shading interp;

view(45,30);

xlabel('x (mm)');

ylabel('y (mm)');

zlabel('Phase (rad)');

title('(c) Reconstructed Phase');

cb = colorbar;

ylabel(cb,'Phase (rad)');

%% =========================================================

% Figure 4: 2D Phase Error Analysis

% (Positions fully aligned with Gaussian model Figure 4)

%

% (a) Phase Error Map

% (b) Absolute Phase Error

% (c) Phase Error Contour

%3D Phase Error Surface

%% =========================================================

figure('Color','w');

%% ---------- (a) Phase Error Map ----------

subplot(1,3,1);

imagesc(x,y,error_map);

axis image;

set(gca,'YDir','normal');

xlabel('x (mm)');

ylabel('y (mm)');

title({ ...

'(a) Phase Reconstruction Error', ...

['RMSE = ',...

num2str(rmse,'%.6f'),...

' rad']});

cb = colorbar;

ylabel(cb,'Error (rad)');

%% ---------- (b) Absolute Error ----------

subplot(1,3,2);

imagesc(x,y,abs_error);

axis image;

set(gca,'YDir','normal');

xlabel('x (mm)');

ylabel('y (mm)');

title('(b) Absolute Phase Error');

cb = colorbar;

ylabel(cb,'Absolute Error (rad)');

%% ---------- (c) Error Contour ----------

subplot(1,3,3);

contourf(Xmm,Ymm,error_map,50,'LineColor','none');

axis image;

xlabel('x (mm)');

ylabel('y (mm)');

title('(c) Phase Error Contour');

cb = colorbar;

ylabel(cb,'Error (rad)');

%% ---------- 3D Error Surface ----------

figure;

surf(Xmm,Ymm,error_map,'EdgeColor','none');

shading interp;

view(45,30);

xlabel('x (mm)');

ylabel('y (mm)');

zlabel('Error (rad)');

title(' 3D Phase Error');

cb = colorbar;

ylabel(cb,'Error (rad)');

set(gcf,'color','w');

set(gca,'color','w');

%% =========================================================

% 26. Center Profile

%% =========================================================

center_true = phi_true(center_row,:);

center_recon = phi_recon(center_row,:);

center_error = error_map(center_row,:);

%% =========================================================

% 27. Diagonal Profile

%% =========================================================

diag_true = diag(phi_true);

diag_recon = diag(phi_recon);

diag_error = diag_true - diag_recon;

diag_coord = linspace(-L0/2,L0/2,N);

%% =========================================================

% Figure 5: Typical Profile Analysis

% (Positions fully aligned with Gaussian model Figure 5)

%

% (a) Horizontal Center-Line Phase

% (b) Horizontal Center-Line Error

% (c) Diagonal Phase Comparison

% (d) Diagonal Phase Error

%% =========================================================

figure('Color','w');

%% ---------- (a) Center-Line Phase ----------

subplot(2,2,1);

plot(x,center_true,'LineWidth',1.5);

hold on;

plot(x,center_recon,'LineWidth',1.2);

grid on;

xlabel('x (mm)');

ylabel('Phase (rad)');

title('(a) Horizontal Center-Line Phase');

legend('Original','Reconstructed','Location','best');

%% ---------- (b) Center-Line Error ----------

subplot(2,2,2);

plot(x,center_error,'LineWidth',1.3);

grid on;

xlabel('x (mm)');

ylabel('Error (rad)');

title('(b) Horizontal Center-Line Error');

%% ---------- (c) Diagonal Phase ----------

subplot(2,2,3);

plot(diag_coord,diag_true,'LineWidth',1.5);

hold on;

plot(diag_coord,diag_recon,'LineWidth',1.2);

grid on;

xlabel('Diagonal Coordinate (mm)');

ylabel('Phase (rad)');

title('(c) Diagonal Phase Comparison');

legend('Original','Reconstructed','Location','best');

%% ---------- (d) Diagonal Error ----------

subplot(2,2,4);

plot(diag_coord,diag_error,'LineWidth',1.3);

grid on;

xlabel('Diagonal Coordinate (mm)');

ylabel('Error (rad)');

title('(d) Diagonal Phase Error');

%% =========================================================

% Figure 6: Error Statistics and Correlation

% (Positions fully aligned with Gaussian model Figure 6)

%

% (a) Phase Error Histogram

% (b) RMSE / MAE / Correlation

% (c) Original vs Reconstructed Phase Scatter Plot

%% =========================================================

figure('Color','w');

%% ---------- (a) Error Histogram ----------

subplot(1,3,1);

histogram(error_map(:),100);

grid on;

xlabel('Phase Error (rad)');

ylabel('Pixel Count');

title('(a) Phase Error Histogram');

%% ---------- (b) Statistical Metrics ----------

subplot(1,3,2);

bar([rmse mae corr_value]);

grid on;

set(gca,...

'XTick',1:3,...

'XTickLabel',...

{'RMSE','MAE','Correlation'});

ylabel('Metric Value');

title('(b) Error Statistics');

%% ---------- (c) Scatter Plot ----------

subplot(1,3,3);

scatter( ...

phi_true(:),...

phi_recon(:),...

8,...

'filled');

hold on;

minVal = min(phi_true(:));

maxVal = max(phi_true(:));

plot( ...

[minVal maxVal],...

[minVal maxVal],...

'r',...

'LineWidth',1.5);

grid on;

axis equal;

xlabel('Original Phase (rad)');

ylabel('Reconstructed Phase (rad)');

title({ ...

'(c) Phase Correlation', ...

['Correlation = ',...

num2str(corr_value,'%.6f')]});

legend('Data','Ideal Line','Location','northwest');

%% =========================================================

% 28. Horizontal / Vertical Center-Line Error

%% =========================================================

center_col = round(N/2);

horizontal_error = ...

error_map(center_row,:);

vertical_error = ...

error_map(:,center_col);

%% =========================================================

% 29. Center-Line RMSE

%% =========================================================

horizontal_rmse = ...

sqrt(mean(horizontal_error.^2));

vertical_rmse = ...

sqrt(mean(vertical_error.^2));

%% =========================================================

% Figure 7: Horizontal / Vertical Center-Line Error

% (Positions fully aligned with Gaussian model Figure 7)

%

% (a) Horizontal / Vertical Center-Line Error

% (b) Center-Line RMSE

%% =========================================================

figure('Color','w');

%% ---------- (a) Center-Line Error Comparison ----------

subplot(1,2,1);

plot(x,horizontal_error,'LineWidth',1.5);

hold on;

plot(y,vertical_error,'LineWidth',1.3);

grid on;

xlabel('Position (mm)');

ylabel('Error (rad)');

title('(a) Horizontal / Vertical Center-Line Error');

legend('Horizontal','Vertical','Location','best');

%% ---------- (b) Center-Line RMSE ----------

subplot(1,2,2);

bar([horizontal_rmse,vertical_rmse]);

grid on;

set(gca,...

'XTick',1:2,...

'XTickLabel',...

{'Horizontal','Vertical'});

ylabel('RMSE (rad)');

title('(b) Center-Line RMSE');

%% =========================================================

% 30. Diagonal RMSE

%% =========================================================

diag_rmse = sqrt(mean(diag_error.^2));

%% =========================================================

% 31. Output Results

%% =========================================================

fprintf('\n');

fprintf('=========================================\n');

fprintf('Four-Level Step Model Digital Holography Reconstruction Completed\n');

fprintf('=========================================\n');

fprintf('Fringe period p	= %d pixel\n',p);

fprintf('Spatial carrier frequency f = %.4f pixel^-1\n',f);

fprintf('\n');

fprintf('RMSE	= %.8f rad\n',rmse);

fprintf('MAE	= %.8f rad\n',mae);

fprintf('Correlation Coefficient = %.8f\n',corr_value);

fprintf('\n');

fprintf('Maximum Absolute Error	= %.8f rad\n',max(abs(error_map(:))));

fprintf('Minimum Absolute Error	= %.8f rad\n',min(abs(error_map(:))));

fprintf('\n');

fprintf('Maximum Diagonal Error	= %.8f rad\n',max(abs(diag_error)));

fprintf('Maximum Center-Line Error = %.8f rad\n',max(abs(center_error)));

fprintf('\n');

fprintf('Horizontal Center-Line RMSE = %.8f rad\n',horizontal_rmse);

fprintf('Vertical Center-Line RMSE	= %.8f rad\n',vertical_rmse);

fprintf('\n');

fprintf('=========================================\n');

fprintf('Unified Figure Specification:\n');

fprintf('Spatial Coordinate	= mm\n');

fprintf('Intensity	= a.u.\n');

fprintf('Phase	= rad\n');

fprintf('Phase Error	= rad\n');

fprintf('Spectrum Coordinate	= pixel^{-1}\n');

fprintf('=========================================\n');

% Siemens Star Phase Digital Holography Reconstruction

% Unified unit specification with Gaussian model:

%	Spatial coordinate: mm

%	Intensity: a.u.

%	Phase: rad

%	Phase error: rad

%	Spectrum coordinate: pixel^{-1}

%% =========================================================

clear;

clc;

close all;

%% =========================================================

% 1. Parameter Settings

%% =========================================================

N = 512;

L0 = 4; % Calculation domain size (mm)

dx = L0 / N; % Pixel size (mm)

lambda = 632.8e-6; % Wavelength (mm)

p = 3; % Fringe period (pixel)

f = 1/p; % Spatial carrier frequency (pixel^-1)

a0 = 0.5;

b0 = 0.5;

phi_step = 0.5; % Maximum phase value (rad)

%% =========================================================

% 2. Siemens Star Phase Modeling

%% =========================================================

[xn,yn] = meshgrid(linspace(-1,1,N));

theta = atan2(yn,xn);

r = sqrt(xn.^2 + yn.^2);

mask = r < 0.85;

sector_num = 36;

phi_true = phi_step .* ...

(0.5 + 0.5*cos(sector_num*theta)) .* mask;

phi_true(phi_true < 0) = 0;

%% =========================================================

% 3. Spatial Coordinate Construction

%% =========================================================

x = linspace(-L0/2,L0/2,N);

y = linspace(-L0/2,L0/2,N);

[X,Y] = meshgrid(x,y);

center_row = round(N/2);

center_col = round(N/2);

%% =========================================================

% 4. Generate Reference Fringe

%% =========================================================

[Xp,~] = meshgrid(1:N,1:N);

I_ref = a0 + ...

b0*cos(2*pi*f*Xp);

%% =========================================================

% 5. Generate Simulated Hologram

%% =========================================================

I_holo = a0 + ...

b0*cos(2*pi*f*Xp + phi_true);

%% =========================================================

% 6. Center Row Data

%% =========================================================

ref_center = I_ref(center_row,:);

holo_center = I_holo(center_row,:);

fringe_diff = holo_center - ref_center;

fringe_2D_diff = I_holo - I_ref;

%% =========================================================

% 7. Fringe Difference Statistics

%% =========================================================

fprintf('\n');

fprintf('=========================================\n');

fprintf('Siemens Star Fringe Difference Analysis\n');

fprintf('=========================================\n');

fprintf('Maximum fringe difference in center row = %.8f\n', ...

max(abs(fringe_diff)));

fprintf('Maximum 2D fringe difference	= %.8f\n', ...

max(abs(fringe_2D_diff(:))));

fprintf('Mean 2D fringe difference	= %.8f\n', ...

mean(abs(fringe_2D_diff(:))));

fprintf('=========================================\n');

%% =========================================================

% Figure 1: Fringe Generation and Difference Analysis

%

% Fully corresponding to Gaussian model Figure 1

%

% (a) Reference Fringe

% (b) Simulated Hologram

% (c) 2D Fringe Difference

% (d) Center-Line Fringe Difference

% (e) Center-Line Fringe Comparison

% (f) Center-Region Enlargement

%% =========================================================

figure('Color','w');

%% ---------- (a) Reference Fringe ----------

subplot(2,3,1);

imagesc(x,y,I_ref);

axis image;

set(gca,'YDir','normal');

xlabel('x (mm)');

ylabel('y (mm)');

title('(a) Reference Fringe');

cb = colorbar;

ylabel(cb,'Intensity (a.u.)');

%% ---------- (b) Simulated Hologram ----------

subplot(2,3,2);

imagesc(x,y,I_holo);

axis image;

set(gca,'YDir','normal');

xlabel('x (mm)');

ylabel('y (mm)');

title('(b) Simulated Hologram');

cb = colorbar;

ylabel(cb,'Intensity (a.u.)');

%% ---------- (c) 2D Fringe Difference ----------

subplot(2,3,3);

imagesc(x,y,fringe_2D_diff);

axis image;

set(gca,'YDir','normal');

xlabel('x (mm)');

ylabel('y (mm)');

title('(c) 2D Fringe Difference');

cb = colorbar;

ylabel(cb,'\Delta I (a.u.)');

%% ---------- (d) Center-Line Fringe Difference ----------

subplot(2,3,4);

plot(x,fringe_diff,...

'LineWidth',1.3);

grid on;

xlabel('x (mm)');

ylabel('\Delta I (a.u.)');

title('(d) Center-Line Fringe Difference');

xlim([min(x) max(x)]);

%% ---------- (e) Center-Line Fringe Comparison ----------

subplot(2,3,5);

plot(x,ref_center,...

'LineWidth',1.5);

hold on;

plot(x,holo_center,...

'LineWidth',1.2);

grid on;

xlabel('x (mm)');

ylabel('Intensity (a.u.)');

title('(e) Center-Line Fringe Comparison');

legend('Reference','Hologram',...

'Location','best');

xlim([min(x) max(x)]);

%% ---------- (f) Center-Region Enlargement ----------

subplot(2,3,6);

plot(x,fringe_diff,...

'LineWidth',1.3);

xlim([-0.6 0.6]);

grid on;

xlabel('x (mm)');

ylabel('\Delta I (a.u.)');

title('(f) Center-Region Enlargement');

%% =========================================================

% 8. FFT Spectrum Calculation

%% =========================================================

F = fft2(I_holo);

F_shift = fftshift(F);

Spectrum = log(1 + abs(F_shift));

Spectrum = Spectrum ./ max(Spectrum(:));

%% =========================================================

% 9. +1 Order Spectrum Position

%% =========================================================

cx = 427;

cy = 256;

%% =========================================================

% 10. Gaussian Frequency Domain Filter Window

%

% Keep all original parameters unchanged

%% =========================================================

sigma_x = 55;

sigma_y = 55;

[u,v] = meshgrid(1:N,1:N);

filter = exp( ...

-((u-cx).^2/(2*sigma_x^2) + ...

(v-cy).^2/(2*sigma_y^2)));

%% =========================================================

% 11. Frequency Domain Filtering

%% =========================================================

F_filter = F_shift .* filter;

Spec_filter = log(1 + abs(F_filter));

Spec_filter = ...

Spec_filter ./ max(Spec_filter(:));

%% =========================================================

% 12. Frequency Coordinate

%% =========================================================

fx = (-N/2:N/2-1)/N;

fy = (-N/2:N/2-1)/N;

%% =========================================================

% Figure 2: Frequency Domain Filtering Process

%

% Fully corresponding to Gaussian model Figure 2

%

% (a) Original Hologram Spectrum

% (b) Gaussian Frequency Domain Filter Window

% (c) Filtered Spectrum

%% =========================================================

figure('Color','w');

%% ---------- (a) Hologram Spectrum ----------

subplot(1,3,1);

imagesc(fx,fy,Spectrum);

axis image;

set(gca,'YDir','normal');

xlabel('f_x (pixel^{-1})');

ylabel('f_y (pixel^{-1})');

title('(a) Hologram Spectrum');

cb = colorbar;

ylabel(cb,'Normalized Magnitude');

%% ---------- (b) Gaussian Filtering Window ----------

subplot(1,3,2);

imagesc(fx,fy,filter);

axis image;

set(gca,'YDir','normal');

xlabel('f_x (pixel^{-1})');

ylabel('f_y (pixel^{-1})');

title('(b) Gaussian Filtering Window');

cb = colorbar;

ylabel(cb,'Transmission');

%% ---------- (c) Filtered Spectrum ----------

subplot(1,3,3);

imagesc(fx,fy,Spec_filter);

axis image;

set(gca,'YDir','normal');

xlabel('f_x (pixel^{-1})');

ylabel('f_y (pixel^{-1})');

title('(c) Filtered Spectrum');

cb = colorbar;

ylabel(cb,'Normalized Magnitude');

%% =========================================================

% 13. Spectrum Shift

%% =========================================================

F_shifted = circshift( ...

F_filter,...

[0 -(cx-N/2)]);

%% =========================================================

% 14. Reconstruct Complex Amplitude

%% =========================================================

U = ifft2(ifftshift(F_shifted));

%% =========================================================

% 15. Construct Reference Complex Field

%% =========================================================

U_ref = exp(1j*2*pi*f*Xp);

%% =========================================================

% 16. Complex Conjugate Multiplication

%% =========================================================

U_phase = U .* conj(U_ref);

%% =========================================================

% 17. Extract Wrapped Phase

%% =========================================================

phi_wrapped = angle(U_phase);

%% =========================================================

% 18. DCT Global Phase Unwrapping

%% =========================================================

gx = diff(phi_wrapped,1,2);

gy = diff(phi_wrapped,1,1);

%% ---------- 2pi Normalization ----------

gx = atan2(sin(gx),cos(gx));

gy = atan2(sin(gy),cos(gy));

%% =========================================================

% 19. Construct Divergence Field

%% =========================================================

rho = zeros(N);

%% ---------- x direction ----------

rho(:,2:end-1) = ...

rho(:,2:end-1) + ...

gx(:,2:end) - gx(:,1:end-1);

%% ---------- y direction ----------

rho(2:end-1,:) = ...

rho(2:end-1,:) + ...

gy(2:end,:) - gy(1:end-1,:);

%% =========================================================

% 20. Neumann Boundary Condition

%% =========================================================

rho(:,1) = gx(:,1);

rho(:,end) = -gx(:,end);

rho(1,:) = rho(1,:) + gy(1,:);

rho(end,:) = rho(end,:) - gy(end,:);

%% =========================================================

% 21. DCT Solve Poisson Equation

%% =========================================================

rho_dct = dct2(rho);

[m,n] = meshgrid(0:N-1,0:N-1);

denominator = ...

2*cos(pi*m/N) + ...

2*cos(pi*n/N) - 4;

denominator(1,1) = 1;

phi_dct = rho_dct ./ denominator;

phi_dct(1,1) = 0;

%% =========================================================

% 22. Inverse DCT Recover Continuous Phase

%% =========================================================

phi_recon = idct2(phi_dct);

%% =========================================================

% 23. Remove Phase Tilt from Reconstructed Phase

%% =========================================================

[Xfit,Yfit] = meshgrid(1:N);

A = [Xfit(:) ...

Yfit(:) ...

ones(N*N,1)];

coef = A \ phi_recon(:);

plane = ...

coef(1)*Xfit + ...

coef(2)*Yfit + ...

coef(3);

phi_recon = phi_recon - plane;

%% =========================================================

% 24. Remove Background Offset

%% =========================================================

background = mean(phi_recon(~mask));

phi_recon = phi_recon - background;

%% =========================================================

% Figure 3: Phase Reconstruction Results

%

% Fully corresponding to Gaussian model Figure 3

%

% (a) Wrapped Phase

% (b) Original Siemens Star Phase

% (c) Reconstructed Siemens Star Phase

%% =========================================================

figure('Color','w');

%% ---------- (a) Wrapped Phase ----------

subplot(1,3,1);

imagesc(x,y,phi_wrapped);

axis image;

set(gca,'YDir','normal');

xlabel('x (mm)');

ylabel('y (mm)');

title('(a) Wrapped Phase');

cb = colorbar;

ylabel(cb,'Phase (rad)');

%% ---------- (b) Original Phase ----------

subplot(1,3,2);

surf(X,Y,phi_true,...

'EdgeColor','none');

shading interp;

view(45,30);

xlabel('x (mm)');

ylabel('y (mm)');

zlabel('Phase (rad)');

title('(b) Original Siemens Star Phase');

cb = colorbar;

ylabel(cb,'Phase (rad)');

%% ---------- (c) Reconstructed Phase ----------

subplot(1,3,3);

surf(X,Y,phi_recon,...

'EdgeColor','none');

shading interp;

view(45,30);

xlabel('x (mm)');

ylabel('y (mm)');

zlabel('Phase (rad)');

title('(c) Reconstructed Siemens Star Phase');

cb = colorbar;

ylabel(cb,'Phase (rad)');

%% =========================================================

% 25. Phase Error Calculation

%% =========================================================

error_map = phi_true - phi_recon;

abs_error = abs(error_map);

%% =========================================================

% 26. RMSE Calculation

%% =========================================================

rmse = sqrt(mean(error_map(:).^2));

%% =========================================================

% 27. MAE Calculation

%% =========================================================

mae = mean(abs(error_map(:)));

%% =========================================================

% 28. Correlation Coefficient

%% =========================================================

R = corrcoef( ...

phi_true(:),...

phi_recon(:));

corr_value = R(1,2);

%% =========================================================

% 29. Diagonal Profile

%% =========================================================

diag_true = diag(phi_true);

diag_recon = diag(phi_recon);

diag_error = ...

diag_true - diag_recon;

diag_coord = ...

linspace(-L0/2,L0/2,N);

%% =========================================================

% 30. Horizontal Center-Line Error

%% =========================================================

horizontal_error = ...

error_map(center_row,:);

%% =========================================================

% 31. Vertical Center-Line Error

%% =========================================================

vertical_error = ...

error_map(:,center_col);

%% =========================================================

% 32. Center-Line RMSE

%% =========================================================

horizontal_rmse = ...

sqrt(mean(horizontal_error.^2));

vertical_rmse = ...

sqrt(mean(vertical_error.^2));

%% =========================================================

% 33. Diagonal RMSE

%% =========================================================

diag_rmse = ...

sqrt(mean(diag_error.^2));

%% =========================================================

% Figure 4: 2D Phase Error Analysis

%

% Fully corresponding to Gaussian model Figure 4

%

% (a) Phase Reconstruction Error

% (b) Absolute Phase Error

% (c) Phase Error Contour

% 3D Phase Error Surface

%% =========================================================

figure('Color','w');

%% ---------- (a) Phase Error Map ----------

subplot(1,3,1);

imagesc(x,y,error_map);

axis image;

set(gca,'YDir','normal');

xlabel('x (mm)');

ylabel('y (mm)');

title({ ...

'(a) Phase Reconstruction Error', ...

['RMSE = ',...

num2str(rmse,'%.6f'),...

' rad']});

cb = colorbar;

ylabel(cb,'Error (rad)');

%% ---------- (b) Absolute Phase Error ----------

subplot(1,3,2);

imagesc(x,y,abs_error);

axis image;

set(gca,'YDir','normal');

xlabel('x (mm)');

ylabel('y (mm)');

title('(b) Absolute Phase Error');

cb = colorbar;

ylabel(cb,'Absolute Error (rad)');

%% ---------- (c) Phase Error Contour ----------

subplot(1,3,3);

contourf(X,Y,error_map,50,...

'LineColor','none');

axis image;

set(gca,'YDir','normal');

xlabel('x (mm)');

ylabel('y (mm)');

title('(c) Phase Error Contour');

cb = colorbar;

ylabel(cb,'Error (rad)');

%% ---------- 3D Phase Error ----------

figure;

surf(X,Y,error_map,...

'EdgeColor','none');

shading interp;

view(45,30);

xlabel('x (mm)');

ylabel('y (mm)');

zlabel('Error (rad)');

title('(c) 3D Phase Error');

cb = colorbar;

ylabel(cb,'Error (rad)');

set(gcf,'color','w');

set(gca,'color','w');

%% =========================================================

% Figure 5: Typical Profile Analysis

%

% Fully corresponding to Gaussian model Figure 5

%

% (a) Horizontal Center-Line Original / Reconstructed Phase

% (b) Horizontal Center-Line Error

% (c) Diagonal Original / Reconstructed Phase

% (d) Diagonal Error

%% =========================================================

center_true = ...

phi_true(center_row,:);

center_recon = ...

phi_recon(center_row,:);

center_error = ...

error_map(center_row,:);

figure('Color','w');

%% ---------- (a) Center-Line Phase ----------

subplot(2,2,1);

plot(x,center_true,...

'LineWidth',1.5);

hold on;

plot(x,center_recon,...

'LineWidth',1.2);

grid on;

xlabel('x (mm)');

ylabel('Phase (rad)');

title('(a) Horizontal Center-Line Phase');

legend('Original','Reconstructed',...

'Location','best');

xlim([min(x) max(x)]);

%% ---------- (b) Center-Line Error ----------

subplot(2,2,2);

plot(x,center_error,...

'LineWidth',1.3);

grid on;

xlabel('x (mm)');

ylabel('Error (rad)');

title('(b) Horizontal Center-Line Error');

xlim([min(x) max(x)]);

%% ---------- (c) Diagonal Phase ----------

subplot(2,2,3);

plot(diag_coord,diag_true,...

'LineWidth',1.5);

hold on;

plot(diag_coord,diag_recon,...

'LineWidth',1.2);

grid on;

xlabel('Diagonal Coordinate (mm)');

ylabel('Phase (rad)');

title('(c) Diagonal Phase Comparison');

legend('Original','Reconstructed',...

'Location','best');

xlim([min(diag_coord) max(diag_coord)]);

%% ---------- (d) Diagonal Error ----------

subplot(2,2,4);

plot(diag_coord,diag_error,...

'LineWidth',1.3);

grid on;

xlabel('Diagonal Coordinate (mm)');

ylabel('Error (rad)');

title('(d) Diagonal Phase Error');

xlim([min(diag_coord) max(diag_coord)]);

%% =========================================================

% Figure 6: Error Statistics and Correlation

%

% Fully corresponding to Gaussian model Figure 6

%

% (a) Phase Error Histogram

% (b) RMSE / MAE / Correlation

% (c) Original vs Reconstructed Phase Scatter Plot

%% =========================================================

figure('Color','w');

%% ---------- (a) Error Histogram ----------

subplot(1,3,1);

histogram(error_map(:),...

100,...

'Normalization','probability');

grid on;

xlabel('Phase Error (rad)');

ylabel('Probability');

title({ ...

'(a) Phase Error Histogram', ...

['RMSE = ',num2str(rmse,'%.6f'),...

' rad']});

%% ---------- (b) Statistical Metrics ----------

subplot(1,3,2);

bar([rmse mae corr_value]);

grid on;

set(gca,...

'XTick',1:3,...

'XTickLabel',...

{'RMSE','MAE','Correlation'});

ylabel('Metric Value');

title('(b) Error Statistics');

%% ---------- (c) Scatter Plot ----------

subplot(1,3,3);

scatter( ...

phi_true(:),...

phi_recon(:),...

8,...

'filled');

hold on;

minVal = min(phi_true(:));

maxVal = max(phi_true(:));

plot( ...

[minVal maxVal],...

[minVal maxVal],...

'LineWidth',1.5);

grid on;

axis equal;

xlabel('Original Phase (rad)');

ylabel('Reconstructed Phase (rad)');

title({ ...

'(c) Phase Correlation', ...

['Correlation = ',...

num2str(corr_value,'%.6f')]});

legend('Data','Ideal Line',...

'Location','northwest');

%% =========================================================

% Figure 7: Horizontal / Vertical Center-Line Error

%

% Fully corresponding to Gaussian model Figure 7

%

% (a) Horizontal / Vertical Center-Line Error

% (b) Center-Line RMSE

%% =========================================================

figure('Color','w');

%% ---------- (a) Center-Line Error Comparison ----------

subplot(1,2,1);

plot(x,horizontal_error,...

'LineWidth',1.5);

hold on;

plot(y,vertical_error,...

'LineWidth',1.3);

grid on;

xlabel('Position (mm)');

ylabel('Error (rad)');

title('(a) Horizontal / Vertical Center-Line Error');

legend('Horizontal','Vertical',...

'Location','best');

xlim([min(x) max(x)]);

%% ---------- (b) Center-Line RMSE ----------

subplot(1,2,2);

bar([ ...

horizontal_rmse,...

vertical_rmse]);

grid on;

set(gca,...

'XTick',1:2,...

'XTickLabel',...

{'Horizontal','Vertical'});

ylabel('RMSE (rad)');

title('(b) Center-Line RMSE');

%% =========================================================

% 34. Final Result Output

%% =========================================================

fprintf('\n');

fprintf('=========================================\n');

fprintf('Siemens Star Digital Holography Reconstruction Completed\n');

fprintf('=========================================\n');

fprintf('Image size	= %d × %d pixel\n',N,N);

fprintf('Calculation domain	= %.4f × %.4f mm^2\n',...

L0,L0);

fprintf('Pixel size	= %.6f mm/pixel\n',...

dx);

fprintf('Fringe period	= %d pixel\n',...

p);

fprintf('Spatial carrier frequency = %.6f pixel^-1\n',...

f);

fprintf('Maximum phase value = %.6f rad\n',...

max(phi_true(:)));

fprintf('\n');

fprintf('RMSE	= %.8f rad\n',...

rmse);

fprintf('MAE	= %.8f rad\n',...

mae);

fprintf('Correlation	= %.8f\n',...

corr_value);

fprintf('\n');

fprintf('Maximum absolute error = %.8f rad\n',...

max(abs_error(:)));

fprintf('Minimum absolute error = %.8f rad\n',...

min(abs_error(:)));

fprintf('\n');

fprintf('Diagonal RMSE	= %.8f rad\n',...

diag_rmse);

fprintf('Maximum diagonal error = %.8f rad\n',...

max(abs(diag_error)));

fprintf('\n');

fprintf('Horizontal center-line RMSE = %.8f rad\n',...

horizontal_rmse);

fprintf('Vertical center-line RMSE	= %.8f rad\n',...

vertical_rmse);

fprintf('Maximum horizontal center-line error = %.8f rad\n',...

max(abs(horizontal_error)));

fprintf('Maximum vertical center-line error	= %.8f rad\n',...

max(abs(vertical_error)));

fprintf('\n');

fprintf('=========================================\n');

fprintf('Unified Figure Unit Specification:\n');

fprintf('Spatial Coordinate	= mm\n');

fprintf('Intensity	= a.u.\n');

fprintf('Phase	= rad\n');

fprintf('Phase Error	= rad\n');

fprintf('Spectrum Coordinate = pixel^{-1}\n');

fprintf('=========================================\n');
