# Supplementary material for: Controlled numerical generation and reconstruction of off-axis digital holograms with object-specific frequency-domain filtering
Source: PLoS One. 2026 Oct 22;21(10):e0360375. doi: 10.1371/journal.pone.0360375 (PMC13690232; doi:10.1371/journal.pone.0360375)
Supplement: Supplementary file 3 [file pone.0360375.s003.doc]

**Response to the Academic Editor and Reviewers**
**Journal:** PLOS ONE

Dear Academic Editor and Reviewers,

We sincerely thank the Academic Editor and the Reviewers for their careful evaluation of our manuscript and for providing valuable comments and constructive suggestions. We have carefully considered all the comments and have made corresponding revisions to improve the clarity, consistency, presentation quality, and reproducibility of the manuscript.

In particular, we have comprehensively revised the figure layouts, standardized the mathematical expressions and parameter definitions, and carefully checked the supplementary code to eliminate untranslated Chinese characters and formatting-related errors.

Our detailed responses to each comment are provided below. All revisions have been carefully checked to ensure consistency with the requirements of the journal.

**Response to the Academic Editor**

**Comment 1: Figure Layout and Subfigure Arrangement**

**Editor's Comment:**

The layouts of all figures need to be further adjusted to comply with the publication standards of PLOS ONE. The current arrangement of subfigures is inappropriate, resulting in substantial unnecessary blank space, particularly in the lower-right areas of the figures. Specific requirements include reorganizing the subfigures in Figures 1–4 and applying consistent alignment, spacing, and grouping principles to all remaining figures.

**Response:**

Thank you for this valuable suggestion. We agree that the original figure layouts did not adequately satisfy the requirements for consistent alignment, spacing, and visual presentation.

In response, we have comprehensively revised the layouts of all figures according to the following principles:

1. **Consistent subfigure dimensions:** The sizes of subfigures have been standardized to ensure visual consistency throughout each composite figure.
2. **Uniform alignment:** Subfigures have been carefully aligned both horizontally and vertically to avoid irregular positioning.
3. **Consistent spacing:** The spacing between subfigures has been adjusted to maintain a uniform layout while reducing unnecessary gaps.
4. **Grouping by test object:** Subfigures corresponding to the same test object have been arranged according to a consistent grouping strategy.
5. **Reduction of unnecessary white space:** The overall figure layouts have been optimized to minimize redundant blank areas, particularly in the lower-right regions.

These revisions were implemented to improve the readability and visual consistency of the figures while better meeting the figure presentation requirements of PLOS ONE.

**Comment 1.1: Figure 1**

**Editor's Comment:**

Figure 1 may be arranged using a six-row layout, with every two rows corresponding to one type of test object: the Gaussian sample, the four-level step sample, and the Siemens star sample. All subfigures should be strictly aligned. For example, subfigures (a), (b), and (e) have the same scale, and therefore these three subfigures can be placed in the first row, with the remaining three subfigures placed in the second row. All subfigures should maintain consistent dimensions and alignment.

**Response:**

Thank you for providing a specific and practical suggestion regarding the arrangement of Figure 1.

We have reorganized Figure 1 according to the recommended six-row layout. The three test objects, namely the Gaussian sample, the four-level step sample, and the Siemens star sample, are now arranged using a consistent two-row structure for each test object.

During the rearrangement, we carefully considered the scale, dimensions, and spatial correspondence of the subfigures. Subfigures with consistent scale characteristics have been placed in corresponding positions, while the dimensions of all subfigures have been standardized.

Furthermore, the alignment of the subfigures has been adjusted to ensure consistent horizontal and vertical positioning. Unnecessary blank space has also been reduced to improve the overall compactness and readability of the figure.

The revised Figure 1 now follows a more consistent and structured arrangement, facilitating comparison among the three test objects.

**Location of Revision:** Revised Figure 1.

**Comment 1.2: Figure 2**

**Editor's Comment:**

All subfigures in Figure 2 should use the same scale. Only one subfigure needs to display the scale bar. The spacing between all subfigures should be reduced and maintained consistently.

**Response:**

We appreciate this suggestion regarding the standardization of scale bars and subfigure spacing.

In the revised Figure 2, all subfigures share the same scale. To avoid unnecessary repetition and visual clutter, the scale bar is retained in only one representative subfigure, while the other subfigures use the same scale reference.

In addition, the spacing between adjacent subfigures has been reduced and standardized. The margins surrounding the composite figure have also been adjusted to minimize unnecessary blank space while maintaining sufficient separation between individual subfigures.

These modifications improve the visual consistency of Figure 2 and facilitate direct comparison among the different test objects.

**Location of Revision:** Revised Figure 2.

**Comment 1.3: Figure 3**

**Editor's Comment:**

The alignment logic for subfigures corresponding to each test object in Figure 3 should be fully consistent with that used for other subfigures of the same type.

**Response:**

Thank you for this comment.

We have revised Figure 3 to ensure that the alignment of subfigures corresponding to each test object follows a consistent layout principle. Subfigures representing the same type of test object are now arranged using standardized dimensions, alignment positions, and inter-subfigure spacing.

The arrangement has been reviewed to ensure that the corresponding subfigures of the Gaussian sample, four-level step sample, and Siemens star sample follow the same organizational logic. This revision improves the consistency of the figure structure and makes comparisons between different test objects more straightforward.

**Location of Revision:** Revised Figure 3.

**Comment 1.4: Figure 4**

**Editor's Comment:**

The subfigures presenting the 3D error results may be moved to the supplementary materials. The remaining subfigures should be reorganized according to the unified layout standards described above.

**Response:**

We thank the Editor for this suggestion.

Following this recommendation, we have reconsidered the organization of the 3D error visualization results. The relevant 3D error subfigures have been separated from the main figure and incorporated into the supplementary materials, thereby reducing the visual complexity of the main text.

The remaining subfigures in Figure 4 have been reorganized according to the unified layout principles applied throughout the manuscript, including consistent subfigure dimensions, alignment, spacing, and grouping of the same type of test object.

This revision allows the main figure to focus on the essential reconstruction and error analysis results while retaining the detailed 3D error visualizations in the supplementary materials.

**Location of Revision:** Revised Figure 4 and Supplementary Materials.

**Comment 1.5: Remaining Figures**

**Editor's Comment:**

All remaining figures should follow the core principles of consistent alignment, uniform spacing, and grouping of subfigures according to their type.

**Response:**

We fully agree with this recommendation.

In addition to Figures 1–4, we have reviewed and revised the layouts of all remaining figures in the manuscript. The following standards have been applied consistently:

- Uniform subfigure dimensions.
- Consistent horizontal and vertical alignment.
- Uniform spacing between adjacent subfigures.
- Grouping of subfigures according to the corresponding test object or analysis category.
- Reduction of unnecessary margins and blank areas.
- Consistent placement of labels, scale bars, and other graphical annotations.

A comprehensive visual inspection was conducted to identify and correct inconsistencies in the arrangement of subfigures.

These revisions improve the overall presentation quality and consistency of the manuscript figures.

**Location of Revision:** All revised figures in the manuscript and supplementary materials.

**Comment 2: Mathematical Formulas and Parameter Definitions**

**Editor's Comment:**

All mathematical expressions should be numbered consistently, and the corresponding equation numbers should be explicitly cited in the main text. Some parameters are insufficiently explained when first introduced and should be clearly and completely defined at their first occurrence. In addition, certain mathematical expressions lack accompanying explanations and punctuation marks, such as those appearing on page 17, lines 4 and 8 from the bottom.

**Response:**

We sincerely thank the Editor for identifying these issues concerning mathematical notation, parameter definitions, and the presentation of equations.

We have systematically reviewed the mathematical expressions throughout the manuscript and made the following revisions.

**2.1 Standardization of Equation Numbering**

All major mathematical expressions requiring independent reference have been assigned consistent equation numbers.

The corresponding equation numbers have also been explicitly cited in the main text wherever the equations are introduced, explained, or subsequently referenced. This ensures a clear connection between the mathematical expressions and their associated descriptions.

**2.2 Improvement of Parameter Definitions**

We have carefully checked the first occurrence of each important parameter in the manuscript.

Where necessary, additional explanations have been provided to clarify:

- The physical meaning of each parameter.
- The corresponding units.
- The role of each parameter in the mathematical model.
- The relationship between the parameter and the experimental or simulation setup.

These definitions have been added at the first relevant occurrence of each parameter to improve the clarity and reproducibility of the proposed method.

**2.3 Revision of Mathematical Expressions and Punctuation**

We have revised the mathematical expressions identified by the Editor, including those located on page 17, fourth and eighth lines from the bottom.

The associated explanatory text and punctuation have been supplemented to ensure that each mathematical expression is properly integrated into the surrounding paragraph. We have also reviewed the consistency of mathematical symbols, notation, and explanatory statements throughout the manuscript.

These revisions improve the readability, logical continuity, and formal presentation of the mathematical content.

**Location of Revision:** Relevant mathematical expressions and parameter definitions throughout the manuscript, including the specified section on page 17.

**Comment 3: Supplementary Code Formatting and Untranslated Characters**

**Editor's Comment:**

The code file submitted as supplementary material still contains a considerable number of Chinese characters that were not correctly translated during the formatting conversion process. All such characters should be replaced with English, and the entire supplementary code file should be checked line by line to ensure that no untranslated Chinese characters, garbled text, or formatting-related abnormal symbols remain.

**Response:**

Thank you for pointing out this issue in the supplementary code.

We recognize that untranslated Chinese characters and formatting-related inconsistencies may affect the readability, accessibility, and reproducibility of the supplementary material, particularly for an international readership.

In response, we have conducted a comprehensive review of the supplementary code file. The following revisions have been performed:

1. **Translation of Chinese text:** All Chinese comments, descriptions, labels, and other text within the code have been translated into English.
2. **Removal of abnormal characters:** Formatting-related garbled characters and abnormal symbols introduced during the document conversion process have been identified and corrected.
3. **Line-by-line inspection:** The supplementary code has been reviewed line by line to identify any remaining untranslated text or formatting inconsistencies.
4. **Consistency of code annotations:** Comments, variable descriptions, and explanatory statements have been checked to ensure consistent English terminology.
5. **Code integrity check:** The revised code has been reviewed to ensure that the text-formatting modifications do not unintentionally affect the structure or execution of the original code.

We have also performed a final inspection of the supplementary code to minimize the possibility of residual untranslated characters or conversion-related formatting errors.

The revised supplementary code has been prepared for resubmission together with the revised manuscript.

**Location of Revision:** Revised Supplementary Code File.

**Concluding Statement**

We sincerely appreciate the Academic Editor's and Reviewers' careful assessment of our manuscript and their constructive recommendations.

In response to the comments, we have revised the figure layouts, standardized the mathematical expressions and parameter definitions, and comprehensively checked the supplementary code. These revisions were undertaken to improve the clarity, consistency, presentation quality, and reproducibility of the manuscript.

We believe that the revised manuscript and supplementary materials have been substantially improved as a result of these changes. We hope that the revisions adequately address the concerns raised during the review process.

Thank you again for your time and consideration.

**Sincerely,**
Xiaolong Shi
On behalf of all authors
