# Supplementary material for: Controlled numerical generation and reconstruction of off-axis digital holograms with object-specific frequency-domain filtering
Source: PLoS One. 2026 Oct 22;21(10):e0360375. doi: 10.1371/journal.pone.0360375 (PMC13690232; doi:10.1371/journal.pone.0360375)
Supplement: Supplementary file 2 [file pone.0360375.s002.docx]

**Response to Reviewer**

Dear Editor and Reviewer,

We sincerely thank the reviewer for the careful evaluation of our manuscript and for the constructive comments. We agree that the original manuscript did not sufficiently clarify the intended role of the simulated holograms, did not adequately discuss the limitations of the synthetic phase models, and did not meet the expected standards for figure presentation and in-text citation organization.

We have therefore substantially revised the manuscript. In particular, we have clarified that the present work is intended to establish a **controlled numerical benchmark for hologram generation, frequency-domain filtering, phase unwrapping, and reconstruction evaluation**, rather than to claim that the current examples constitute a complete deep-learning training dataset or a comprehensive representation of experimental holographic measurements.

We have also reorganized the figures into seven multi-panel composite figures, unified coordinate-axis labels and physical units, revised the figure captions, strengthened the discussion of the limitations of the numerical models, and systematically incorporated references at the corresponding positions in the main text.

Our detailed responses are provided below.

**Comment 1**

**Reviewer comment:**

The authors state that the research objective is to generate synthetic holograms for “deep-learning holographic algorithms,” but they do not specify what tasks these deep-learning algorithms are intended to perform. If the purpose is to train deep-learning networks for phase unwrapping or phase recovery, the generated holograms are too simple, because the authors can already recover the correct phase using conventional methods. In this case, the necessity of deep learning is unclear. Even if the holograms are intended for another task, synthetic hologram datasets generally require thousands of samples, whereas the manuscript presents only a few simple examples.

**Response**

We thank the reviewer for identifying an important ambiguity in the original manuscript. We agree that the original wording could incorrectly suggest that the present study was intended to provide a complete training dataset for deep-learning-based phase reconstruction.

This was not our intended claim. The purpose of the present study is more limited and specific: **to establish a reproducible numerical benchmark in which the ground-truth phase is analytically known and the effects of hologram generation, spectral filtering, phase extraction, and phase unwrapping can be quantitatively evaluated.**

We have therefore revised the manuscript to explicitly distinguish between a **controlled benchmark dataset** and a **large-scale deep-learning training dataset**.

The revised manuscript now states that:

“Rather than claiming that a small set of analytical examples constitutes a complete training corpus for deep learning, the present work establishes reproducible benchmark cases for evaluating hologram generation, frequency-domain filtering, phase unwrapping, and reconstruction accuracy.”

This clarification has been added to the **Introduction**.

We have also explicitly stated that the current synthetic cases are intended primarily for:

1. verification of hologram-generation procedures;
2. baseline evaluation of phase reconstruction algorithms;
3. quantitative comparison with known analytical ground truth; and
4. providing controlled baseline cases before more complex experimental or learning-based datasets are introduced.

We agree with the reviewer that a small number of analytical examples should **not** be presented as sufficient for training a general-purpose deep-learning model. Accordingly, we have removed language that could imply that the present study provides a complete deep-learning training corpus.

We have also added a limitation statement explaining that systematic development of larger and more diverse datasets will be necessary for future deep-learning applications. The revised manuscript now explicitly identifies the development of larger datasets as future work.

Therefore, the role of deep learning in the revised manuscript is no longer presented as a prerequisite for solving the simple benchmark cases. Instead, the proposed numerical framework provides **ground-truth-controlled baseline data against which future learning-based reconstruction methods can be evaluated**.

This change also makes the scientific purpose of the current study more precise: the contribution is not to demonstrate that deep learning is necessary for these simple analytical objects, but to establish a reproducible numerical reference framework that can subsequently be used to evaluate more sophisticated algorithms.

**Changes made:**

1. Revised the **Abstract** to define the dataset as a controlled benchmark rather than a complete deep-learning corpus.
2. Revised the **Introduction** to clarify the intended use of the simulated holograms.
3. Added an explicit discussion of the limitations of the current dataset.
4. Revised the **Conclusion and Future Work** to state that larger and more diverse datasets are required for future deep-learning-based phase reconstruction.

**Comment 2**

**Reviewer comment:**

The datasets generated by the proposed method are much simpler than experimentally acquired holograms in metrology or microscopy. All targets are pure two-dimensional phase objects, without amplitude modulation or three-dimensional structure.

**Response**

We agree with the reviewer.

The present models are deliberately simplified and represent **controlled two-dimensional phase-only objects**. They were selected because the analytical ground-truth phase is exactly known, which allows reconstruction errors to be separated from experimental uncertainties.

We recognize that this simplification limits the direct applicability of the present simulations to real experimental holographic microscopy and metrology. In particular, the current model does not include several important experimental factors, including:

1. amplitude-phase coupling;
2. speckle;
3. optical aberrations;
4. illumination nonuniformity;
5. sensor nonuniformity;
6. multiple scattering;
7. three-dimensional object structure;
8. multiple depth planes; and
9. experimentally measured noise characteristics.

Rather than attempting to conceal these limitations, we have explicitly incorporated them into the revised manuscript.

The revised manuscript now states:

“The present study does not attempt to reproduce every experimental factor, such as amplitude-phase coupling, speckle, aberration, sensor nonuniformity, multiple scattering, or complex three-dimensional specimens. The synthetic cases are therefore treated as controlled benchmark samples rather than as a substitute for experimental data.”

This clarification is important because it changes the interpretation of the three models. They are no longer presented as representative of the full diversity of industrial or biomedical specimens.

**Regarding the Gaussian, four-level step, and Siemens Star models**

We retained the three models because they provide complementary spatial characteristics:

1. **Gaussian phase model:** smooth and continuously varying phase;
2. **four-level step model:** abrupt one-dimensional phase transitions;
3. **Siemens Star model:** radially distributed high-frequency structural boundaries.

Thus, the three models provide progressively different spectral and spatial characteristics while retaining analytically known ground truth.

The Siemens Star is specifically treated as an additional structured numerical target rather than as a replacement for a real experimental calibration specimen.

We have therefore revised the manuscript to avoid statements implying that these models represent the complete phase distributions encountered in industrial inspection or biomedical imaging.

In particular, we removed the unsupported quantitative claim that the benchmark models represent a specified percentage of industrial or biomedical phase distributions. We agree that such a numerical claim would require a dedicated statistical study and corresponding literature evidence.

**Future extension**

We have also added a dedicated limitation/future-work statement indicating that future versions of the framework should incorporate:

1. amplitude modulation;
2. realistic noise;
3. speckle;
4. optical aberration;
5. illumination nonuniformity;
6. multiple scattering;
7. multiple depth planes; and
8. experimentally recorded holograms.

We believe this revision gives a more scientifically appropriate description of the scope of the present work and prevents the numerical benchmark from being interpreted as a complete substitute for experimental holographic datasets.

**Changes made:**

1. Revised the **Introduction** to explicitly distinguish controlled numerical data from experimental holograms.
2. Revised the **Methods** section to describe the Gaussian, four-level step, and Siemens Star models as controlled benchmark objects.
3. Removed unsupported claims concerning the percentage of industrial/biomedical phase distributions represented by the models.
4. Added explicit limitations concerning amplitude, noise, speckle, aberration, multiple scattering, and three-dimensional structure.
5. Expanded the **Future Work** section to describe the planned transition from controlled 2D simulations toward experimentally validated and more diverse holographic datasets.
1. **Comment 3**

**Reviewer comment:**

The presentation quality of the manuscript is poor and does not follow basic scientific-literature conventions. In particular, references to other studies are listed at the end but are not properly cited in the main text. Many statements require supporting references. For example, the statement that the two benchmark phase models cover more than 90% of phase-distribution characteristics in industrial inspection and biomedical imaging requires a corresponding reference. In addition, all figures are presented as independent panels, resulting in too many figures. The resolution is low and the images are highly compressed. Many axes lack names and units.

**Response**

We fully agree with the reviewer that the original presentation did not meet an appropriate level of scientific clarity and consistency.

We have made substantial changes to both the **citation organization** and **figure presentation**.

**3.1 In-text citation organization**

We have reorganized the literature citations so that references are now cited at the relevant positions in the main text rather than being listed only at the end of the manuscript.

In particular, references are now inserted when discussing:

1. the historical development of holography and off-axis holography;
2. digital holography and quantitative phase imaging;
3. phase reconstruction and phase unwrapping;
4. digital holographic microscopy;
5. computational holography and related numerical reconstruction methods;
6. optical aberration and experimental limitations; and
7. deep-learning or learning-based holographic reconstruction.

We also removed statements that could not be adequately supported by the available references.

Most importantly, the previous quantitative statement that the proposed phase models cover “more than 90%” of phase-distribution characteristics in industrial inspection and biomedical imaging has been removed. We agree that such a precise percentage cannot be introduced without a dedicated statistical analysis and appropriate literature support.

The revised text instead states that the models represent **complementary phase characteristics** and are intended as controlled benchmark cases rather than as statistical representations of experimental object populations.

This modification prevents an unsupported numerical generalization from being presented as an established fact.

**3.2 Figure organization**

We agree that the original figure organization resulted in an unnecessarily large number of independent figures.

To address this issue, we reorganized the results into **seven multi-panel composite figures**.

The revised figure structure is:

**Figure 1 — Benchmark phase models**

1. Gaussian phase model;
2. four-level step phase model;
3. Siemens Star phase model;
4. corresponding 2D/3D representations where appropriate.

**Figure 2 — Simulated holograms and fringe modulation**

1. reference fringe;
2. simulated holograms;
3. representative central-line fringe comparison;
4. fringe-difference analysis.

**Figure 3 — Frequency-domain filtering**

1. hologram spectra;
2. filtering windows;
3. filtered spectra for the different phase models.

**Figure 4 — Phase reconstruction**

1. wrapped phase;
2. original phase;
3. reconstructed phase for the different benchmark objects.

**Figure 5 — Representative phase profiles**

1. horizontal center-line comparison;
2. center-line error;
3. diagonal phase comparison;
4. diagonal error.

**Figure 6 — Spatial and statistical error analysis**

1. error maps;
2. absolute error;
3. error surfaces;
4. error distributions/histograms;
5. quantitative reconstruction metrics.

**Figure 7 — Comprehensive reconstruction evaluation**

1. original-versus-reconstructed phase scatter plots;
2. diagonal profiles;
3. horizontal/vertical center-line errors;
4. corresponding quantitative comparisons for the three benchmark cases.

This organization substantially reduces the number of independent figures while retaining the information contained in the original results.

**3.3 Coordinate axes and units**

We have also standardized the coordinate and unit conventions throughout the figures.

The revised manuscript uses:

1. **Spatial coordinates:** x (mm) and y (mm)
2. **Phase:** Phase (rad)
3. **Phase error:** Error (rad)
4. **Intensity:** Intensity (a.u.)
5. **Intensity difference:** ΔI (a.u.)
6. **Spatial frequency:** f_x (pixel⁻¹) and f_y (pixel⁻¹)
7. **Profile coordinate:** Position (mm) where physical coordinates are used.

The corresponding colorbars have also been explicitly labeled with their physical quantities and units.

This standardization was applied consistently to the multi-panel figures so that the axes, colorbars, labels, and captions follow the same format.

**3.4 Figure quality and readability**

We also agree that the original figures were not sufficiently suitable for publication.

The figures have therefore been reorganized as high-resolution composite figures rather than collections of separately compressed images. The multi-panel layout also reduces redundant plotting and allows related results to be compared directly within the same figure.

The figure captions have been rewritten so that every panel is explicitly identified as (a), (b), (c), etc., and the corresponding physical quantities and units are stated in the captions.

We believe that these changes substantially improve the readability, consistency, and publication quality of the figures.

**Additional clarification regarding the scope of the manuscript**

Following the reviewer's comments, we have also reconsidered the overall positioning of the manuscript.

The revised manuscript does **not** claim that the current three analytical models reproduce the full complexity of experimental digital holography. Instead, the study is positioned as a **controlled numerical benchmark**.

The main purpose is to provide:

1. analytically defined phase ground truth;
2. reproducible off-axis hologram generation;
3. controlled spectral characteristics;
4. quantitative evaluation of frequency-domain filtering;
5. quantitative evaluation of phase unwrapping; and
6. baseline error measurements for future experimental and learning-based methods.

This revised positioning is more consistent with the actual scope of the numerical experiments.

The revised manuscript explicitly acknowledges that larger, more diverse datasets and experimental validation will be necessary before the framework can be used for general-purpose deep-learning training or quantitative experimental metrology.

**Summary of major revisions**

In response to the reviewer, the following major changes have been made:

1. The intended role of the synthetic holograms has been clarified.
2. The manuscript no longer presents the current examples as a complete deep-learning training dataset.
3. The limitation of using only two-dimensional phase-only models has been explicitly acknowledged.
4. The absence of amplitude modulation, realistic noise, speckle, aberration, multiple scattering, and 3D structures has been discussed.
5. The unsupported quantitative claim regarding coverage of industrial and biomedical phase distributions has been removed.
6. References have been incorporated into the corresponding positions in the main text.
7. Statements requiring literature support have been revised and cited where appropriate.
8. The original large number of individual figures has been reorganized into seven multi-panel composite figures.
9. Coordinate axes, physical quantities, and units have been standardized across all figures.
10. Figure captions have been rewritten to identify individual panels and their corresponding quantities.
11. The limitations and future development of the numerical framework have been explicitly added to the manuscript.
12. The overall manuscript has been revised to present the work as a reproducible numerical benchmark rather than as a complete substitute for experimental holographic datasets.

We sincerely appreciate the reviewer’s comments. We believe that these revisions have substantially improved the scientific positioning, transparency, reproducibility, and presentation quality of the manuscript.

Thank you again for the constructive suggestions.

Sincerely,

The Authors
