# Supplementary material for: Controlled numerical generation and reconstruction of off-axis digital holograms with object-specific frequency-domain filtering
Source: PLoS One. 2026 Oct 22;21(10):e0360375. doi: 10.1371/journal.pone.0360375 (PMC13690232; doi:10.1371/journal.pone.0360375)
Supplement: Peer review history

# Peer review: Decision Letter 0

**DOI:** 10.1371/journal.pone.0360375.r001

**Authors:** Maddalena Collini

25 Jul 2026

Dear Dr. shi,

Please submit your revised manuscript by Sep 23 2026 11:59PM. If you will need more time than this to complete your revisions, please reply to this message or contact the journal office at plosone@plos.org. When you're ready to submit your revision, log on to https://www.editorialmanager.com/pone/ and select the 'Submissions Needing Revision' folder to locate your manuscript file.

- A letter that responds to each point raised by the academic editor and reviewer(s). You should upload this letter as a separate file labeled 'Response to Reviewers'.
- A marked-up copy of your manuscript that highlights changes made to the original version. You should upload this as a separate file labeled 'Revised Manuscript with Track Changes'.
- An unmarked version of your revised paper without tracked changes. You should upload this as a separate file labeled 'Manuscript'.

If you would like to make changes to your financial disclosure, please include your updated statement in your cover letter. Guidelines for resubmitting your figure files are available below the reviewer comments at the end of this letter.

If applicable, we recommend that you deposit your laboratory protocols in protocols.io to enhance the reproducibility of your results. Protocols.io assigns your protocol its own identifier (DOI) so that it can be cited independently in the future. For instructions see: https://journals.plos.org/plosone/s/submission-guidelines#loc-laboratory-protocols. Additionally, PLOS ONE offers an option for publishing peer-reviewed Lab Protocol articles, which describe protocols hosted on protocols.io. Read more information on sharing protocols at https://plos.org/protocols?utm_medium=editorial-email&utm_source=authorletters&utm_campaign=protocols.

As the corresponding author, your ORCID iD is verified in the submission system and will appear in the published article. PLOS supports the use of ORCID, and we encourage all coauthors to register for an ORCID iD and use it as well. Please encourage your coauthors to verify their ORCID iD within the submission system before final acceptance, as unverified ORCID iDs will not appear in the published article. *Only*  the individual author can complete the verification step; PLOS staff *cannot*  verify ORCID iDs on behalf of authors.

We look forward to receiving your revised manuscript.

Kind regards,

Maddalena Collini, PhD

Academic Editor

PLOS One

Journal Requirements:

When submitting your revision, we need you to address these additional requirements.

1.Please ensure that your manuscript meets PLOS ONE's style requirements, including those for file naming. The PLOS ONE style templates can be found at

https://journals.plos.org/plosone/s/file?id=wjVg/PLOSOne_formatting_sample_main_body.pdf and

https://journals.plos.org/plosone/s/file?id=ba62/PLOSOne_formatting_sample_title_authors_affiliations.pdf

2. PLOS requires an ORCID iD for the corresponding author in Editorial Manager on papers submitted after December 6th, 2016. Please ensure that you have an ORCID iD and that it is validated in Editorial Manager. To do this, go to ‘Update my Information’ (in the upper left-hand corner of the main menu), and click on the Fetch/Validate link next to the ORCID field. This will take you to the ORCID site and allow you to create a new iD or authenticate a pre-existing iD in Editorial Manager.

3. Please note that PLOS One has specific guidelines on code sharing for submissions in which author-generated code underpins the findings in the manuscript. In these cases, we expect all author-generated code to be made available without restrictions upon publication of the work. Please review our guidelines at https://journals.plos.org/plosone/s/materials-and-software-sharing#loc-sharing-code and ensure that your code is shared in a way that follows best practice and facilitates reproducibility and reuse.

4. Thank you for stating the following in your Competing Interests section:

“The author declares no conflict of interest”

Please complete your Competing Interests on the online submission form to state any Competing Interests. If you have no competing interests, please state "The authors have declared that no competing interests exist.", as detailed online in our guide for authors at http://journals.plos.org/plosone/s/submit-now

This information should be included in your cover letter; we will change the online submission form on your behalf.

5. We note that the grant information you provided in the ‘Funding Information’ and ‘Financial Disclosure’ sections do not match.

When you resubmit, please ensure that you provide the correct grant numbers for the awards you received for your study in the ‘Funding Information’ section.

6. When completing the data availability statement of the submission form, you indicated that you will make your data available on acceptance. We strongly recommend all authors decide on a data sharing plan before acceptance, as the process can be lengthy and hold up publication timelines. Please note that, though access restrictions are acceptable now, your entire data will need to be made freely accessible if your manuscript is accepted for publication. This policy applies to all data except where public deposition would breach compliance with the protocol approved by your research ethics board. If you are unable to adhere to our open data policy, please kindly revise your statement to explain your reasoning and we will seek the editor's input on an exemption. Please be assured that, once you have provided your new statement, the assessment of your exemption will not hold up the peer review process.

7. Please upload a new copy of Figures 1-20 as the detail is not clear. Please follow the link for more information:  https://journals.plos.org/plosone/s/figures.

8. We notice that your supplementary figures are uploaded with the file type 'Figure'. Please amend the file type to 'Supporting Information'. Please ensure that each Supporting Information file has a legend listed in the manuscript after the references list.

9. If the reviewer comments include a recommendation to cite specific previously published works, please review and evaluate these publications to determine whether they are relevant and should be cited. There is no requirement to cite these works unless the editor has indicated otherwise.

Additional Editor Comments:

In particular, the figures must be reorganized into multi-panel figures with the required resolution and image quality. They should be grouped according to the logical flow of the results presented in the text. The manuscript text should also be revised to improve clarity and readability. Ensure that all references are properly cited within the text and that all relevant statements are supported by appropriate literature citations.

In addition, carefully consider the suggestions provided by the reviewers to further improve the overall quality of the manuscript. In the response letter accompanying the revised manuscript, clearly explain how each suggestion has been addressed or, where applicable, provide a justification for why a recommendation was not incorporated.

[Note: HTML markup is below. Please do not edit.]

Reviewer's Responses to Questions

**Comments to the Author**

1. Is the manuscript technically sound, and do the data support the conclusions?

Reviewer #1: No

Reviewer #2: Yes

**********

2. Has the statistical analysis been performed appropriately and rigorously?

Reviewer #1: No

Reviewer #2: I Don't Know

**********

3. Have the authors made all data underlying the findings in their manuscript fully available?

The PLOS Data policy

Reviewer #1: No

Reviewer #2: No

**********

4. Is the manuscript presented in an intelligible fashion and written in standard English?

Reviewer #1: No

Reviewer #2: Yes

**********

Reviewer #1: The manuscript aims to present a method for the generation of holograms intended for the training of Deep Learning algorithms for digital holography. The goal of the research is very vaguely stated, the methods and results presented are difficult to comprehend, and the manuscript presentation does not follow even the most basic standards for scientific literature. Given these considerations, i recommend rejecting the manuscript.

To expend my reasoning in more detail:

1 - The authors state that their goal is the generation of synthetic holograms intended for "deep learning holographic algorithms". They do not state what these "deep learning algorithms" are supposed to do with holograms. If the goal is to train deep learning networks for phase unwrapping and retrieval, the holograms computed by the authors are definitely too simplistic, since the authors themselves can retrieve the correct phase through simple methods, rendering the deep learning redundant. Even supposing holograms are to be used for some different unexplained goal, generation of synthetic holograms should provide thousands of datasets, while the authors only show a handful of very simple examples.

2 - Dataset realized through the authors methods seem extremely simple compared to experimental holograms acquired in the metrology or microscopy fields that they seem to target. In particular, all objects are generated as purely two-dimensional phase objects, with no light intensity modulation, and no three-dimensional structure.

3 - The manuscript is very poorly presented, and does not respect very simple conventions of scientific literature. In particular, references to other works are only reported in bulk at the end of the manuscript, and not cited throughout the text. This is a fundamental problem, as many statements throughout the text would benefit from a literature reference in support, and as stated seem unbelievable (e.g. at some point the authors state "The two types of bechmark phase models constructed in this study cover more than 90% of the phase distribution characteristics in industrial inspection and biomedical imaging scenarios". This statement with such a precise numerical estimate would definitely need support from the literature).

The figures are presented as separate single panels in a staggering number, with such a low resolution and high compression that makes most lines and numbers unreadable, and often not reporting names or units on the axes.

Reviewer #2: The ms. treat a practical problem often encountered in digital holography: the problem of the phase unwrapping for stepwise objects. The text is written in clear way but it can be improved. What is really hindering a fruitful reading is the dispersion of the results and the analysis in an amazingly high number of figures for a scientific paper. Moreover, the quality of the figures in the pdf I could download is very poor and in many cases they cannot be inspected for reviewing.

I suggest to re-organize the figures in a smaller number of figures with a few panels per figure, clustering together the figures that pertains to a single concept that the Authors want to convey. And improve the quality of the figures.

For the rest, I suggest to make available the code on a repository as well as the simualated data. This would make the Ms. much more palatable (and citable) for the readers.

**********

what does this mean?). If published, this will include your full peer review and any attached files.

If you choose “no”, your identity will remain anonymous but your review may still be made public.

**Do you want your identity to be public for this peer review?**  For information about this choice, including consent withdrawal, please see our Privacy Policy

Reviewer #1: No

Reviewer #2: **Yes:**  Giuseppe Chirico

**********

[NOTE: If reviewer comments were submitted as an attachment file, they will be attached to this email and accessible via the submission site. Please log into your account, locate the manuscript record, and check for the action link "View Attachments". If this link does not appear, there are no attachment files.]

To ensure your figures meet our technical requirements, please review our figure guidelines: https://journals.plos.org/plosone/s/figures

You may also use PLOS’s free figure tool, NAAS, to help you prepare publication quality figures: https://journals.plos.org/plosone/s/figures#loc-tools-for-figure-preparation.

NAAS will assess whether your figures meet our technical requirements by comparing each figure against our figure specifications.

# Author comment: Author response to Decision Letter 1

**DOI:** 10.1371/journal.pone.0360375.r002

8 Sep 2026

Comment 1

Reviewer comment:

The authors state that the research objective is to generate synthetic holograms for “deep-learning holographic algorithms,” but they do not specify what tasks these deep-learning algorithms are intended to perform. If the purpose is to train deep-learning networks for phase unwrapping or phase recovery, the generated holograms are too simple, because the authors can already recover the correct phase using conventional methods. In this case, the necessity of deep learning is unclear. Even if the holograms are intended for another task, synthetic hologram datasets generally require thousands of samples, whereas the manuscript presents only a few simple examples.

Response

We thank the reviewer for identifying an important ambiguity in the original manuscript. We agree that the original wording could incorrectly suggest that the present study was intended to provide a complete training dataset for deep-learning-based phase reconstruction.

This was not our intended claim. The purpose of the present study is more limited and specific: to establish a reproducible numerical benchmark in which the ground-truth phase is analytically known and the effects of hologram generation, spectral filtering, phase extraction, and phase unwrapping can be quantitatively evaluated.

We have therefore revised the manuscript to explicitly distinguish between a controlled benchmark dataset and a large-scale deep-learning training dataset.

The revised manuscript now states that:

“Rather than claiming that a small set of analytical examples constitutes a complete training corpus for deep learning, the present work establishes reproducible benchmark cases for evaluating hologram generation, frequency-domain filtering, phase unwrapping, and reconstruction accuracy.”

This clarification has been added to the Introduction.

We have also explicitly stated that the current synthetic cases are intended primarily for:

1. verification of hologram-generation procedures;

2. baseline evaluation of phase reconstruction algorithms;

3. quantitative comparison with known analytical ground truth; and

4. providing controlled baseline cases before more complex experimental or learning-based datasets are introduced.

We agree with the reviewer that a small number of analytical examples should not be presented as sufficient for training a general-purpose deep-learning model. Accordingly, we have removed language that could imply that the present study provides a complete deep-learning training corpus.

We have also added a limitation statement explaining that systematic development of larger and more diverse datasets will be necessary for future deep-learning applications. The revised manuscript now explicitly identifies the development of larger datasets as future work.

Therefore, the role of deep learning in the revised manuscript is no longer presented as a prerequisite for solving the simple benchmark cases. Instead, the proposed numerical framework provides ground-truth-controlled baseline data against which future learning-based reconstruction methods can be evaluated.

This change also makes the scientific purpose of the current study more precise: the contribution is not to demonstrate that deep learning is necessary for these simple analytical objects, but to establish a reproducible numerical reference framework that can subsequently be used to evaluate more sophisticated algorithms.

Changes made:

1. Revised the Abstract to define the dataset as a controlled benchmark rather than a complete deep-learning corpus.

2. Revised the Introduction to clarify the intended use of the simulated holograms.

3. Added an explicit discussion of the limitations of the current dataset.

4. Revised the Conclusion and Future Work to state that larger and more diverse datasets are required for future deep-learning-based phase reconstruction.

Comment 2

Reviewer comment:

The datasets generated by the proposed method are much simpler than experimentally acquired holograms in metrology or microscopy. All targets are pure two-dimensional phase objects, without amplitude modulation or three-dimensional structure.

Response

We agree with the reviewer.

The present models are deliberately simplified and represent controlled two-dimensional phase-only objects. They were selected because the analytical ground-truth phase is exactly known, which allows reconstruction errors to be separated from experimental uncertainties.

We recognize that this simplification limits the direct applicability of the present simulations to real experimental holographic microscopy and metrology. In particular, the current model does not include several important experimental factors, including:

1. amplitude-phase coupling;

2. speckle;

3. optical aberrations;

4. illumination nonuniformity;

5. sensor nonuniformity;

6. multiple scattering;

7. three-dimensional object structure;

8. multiple depth planes; and

9. experimentally measured noise characteristics.

Rather than attempting to conceal these limitations, we have explicitly incorporated them into the revised manuscript.

The revised manuscript now states:

“The present study does not attempt to reproduce every experimental factor, such as amplitude-phase coupling, speckle, aberration, sensor nonuniformity, multiple scattering, or complex three-dimensional specimens. The synthetic cases are therefore treated as controlled benchmark samples rather than as a substitute for experimental data.”

This clarification is important because it changes the interpretation of the three models. They are no longer presented as representative of the full diversity of industrial or biomedical specimens.

Regarding the Gaussian, four-level step, and Siemens Star models

We retained the three models because they provide complementary spatial characteristics:

1. Gaussian phase model: smooth and continuously varying phase;

2. four-level step model: abrupt one-dimensional phase transitions;

3. Siemens Star model: radially distributed high-frequency structural boundaries.

Thus, the three models provide progressively different spectral and spatial characteristics while retaining analytically known ground truth.

The Siemens Star is specifically treated as an additional structured numerical target rather than as a replacement for a real experimental calibration specimen.

We have therefore revised the manuscript to avoid statements implying that these models represent the complete phase distributions encountered in industrial inspection or biomedical imaging.

In particular, we removed the unsupported quantitative claim that the benchmark models represent a specified percentage of industrial or biomedical phase distributions. We agree that such a numerical claim would require a dedicated statistical study and corresponding literature evidence.

Future extension

We have also added a dedicated limitation/future-work statement indicating that future versions of the framework should incorporate:

1. amplitude modulation;

2. realistic noise;

3. speckle;

4. optical aberration;

5. illumination nonuniformity;

6. multiple scattering;

7. multiple depth planes; and

8. experimentally recorded holograms.

We believe this revision gives a more scientifically appropriate description of the scope of the present work and prevents the numerical benchmark from being interpreted as a complete substitute for experimental holographic datasets.

Changes made:

1. Revised the Introduction to explicitly distinguish controlled numerical data from experimental holograms.

2. Revised the Methods section to describe the Gaussian, four-level step, and Siemens Star models as controlled benchmark objects.

3. Removed unsupported claims concerning the percentage of industrial/biomedical phase distributions represented by the models.

4. Added explicit limitations concerning amplitude, noise, speckle, aberration, multiple scattering, and three-dimensional structure.

5. Expanded the Future Work section to describe the planned transition from controlled 2D simulations toward experimentally validated and more diverse holographic datasets.

1. Comment 3

Reviewer comment:

The presentation quality of the manuscript is poor and does not follow basic scientific-literature conventions. In particular, references to other studies are listed at the end but are not properly cited in the main text. Many statements require supporting references. For example, the statement that the two benchmark phase models cover more than 90% of phase-distribution characteristics in industrial inspection and biomedical imaging requires a corresponding reference. In addition, all figures are presented as independent panels, resulting in too many figures. The resolution is low and the images are highly compressed. Many axes lack names and units.

Response

We fully agree with the reviewer that the original presentation did not meet an appropriate level of scientific clarity and consistency.

We have made substantial changes to both the citation organization and figure presentation.

3.1 In-text citation organization

We have reorganized the literature citations so that references are now cited at the relevant positions in the main text rather than being listed only at the end of the manuscript.

In particular, references are now inserted when discussing:

1. the historical development of holography and off-axis holography;

2. digital holography and quantitative phase imaging;

3. phase reconstruction and phase unwrapping;

4. digital holographic microscopy;

5. computational holography and related numerical reconstruction methods;

6. optical aberration and experimental limitations; and

7. deep-learning or learning-based holographic reconstruction.

We also removed statements that could not be adequately supported by the available references.

Most importantly, the previous quantitative statement that the proposed phase models cover “more than 90%” of phase-distribution characteristics in industrial inspection and biomedical imaging has been removed. We agree that such a precise percentage cannot be introduced without a dedicated statistical analysis and appropriate literature support.

The revised text instead states that the models represent complementary phase characteristics and are intended as controlled benchmark cases rather than as statistical representations of experimental object populations.

This modification prevents an unsupported numerical generalization from being presented as an established fact.

3.2 Figure organization

We agree that the original figure organization resulted in an unnecessarily large number of independent figures.

To address this issue, we reorganized the results into seven multi-panel composite figures.

The revised figure structure is:

Figure 1 — Benchmark phase models

1. Gaussian phase model;

2. four-level step phase model;

3. Siemens Star phase model;

4. corresponding 2D/3D representations where appropriate.

Figure 2 — Simulated holograms and fringe modulation

1. reference fringe;

2. simulated holograms;

3. representative central-line fringe comparison;

4. fringe-difference analysis.

Figure 3 — Frequency-domain filtering

1. hologram spectra;

2. filtering windows;

3. filtered spectra for the different phase models.

Figure 4 — Phase reconstruction

1. wrapped phase;

2. original phase;

3. reconstructed phase for the different benchmark objects.

Figure 5 — Representative phase profiles

1. horizontal center-line comparison;

2. center-line error;

3. diagonal phase comparison;

4. diagonal error.

Figure 6 — Spatial and statistical error analysis

1. error maps;

2. absolute error;

3. error surfaces;

4. error distributions/histograms;

5. quantitative reconstruction metrics.

Figure 7 — Comprehensive reconstruction evaluation

1. original-versus-reconstructed phase scatter plots;

2. diagonal profiles;

3. horizontal/vertical center-line errors;

4. corresponding quantitative comparisons for the three benchmark cases.

This organization substantially reduces the number of independent figures while retaining the information contained in the original results.

3.3 Coordinate axes and units

We have also standardized the coordinate and unit conventions throughout the figures.

The revised manuscript uses:

1. Spatial coordinates: x (mm) and y (mm)

2. Phase: Phase (rad)

3. Phase error: Error (rad)

4. Intensity: Intensity (a.u.)

5. Intensity difference: ΔI (a.u.)

6. Spatial frequency: f_x (pixel⁻¹) and f_y (pixel⁻¹)

7. Profile coordinate: Position (mm) where physical coordinates are used.

The corresponding colorbars have also been explicitly labeled with their physical quantities and units.

This standardization was applied consistently to the multi-panel figures so that the axes, colorbars, labels, and captions follow the same format.

3.4 Figure quality and readability

We also agree that the original figures were not sufficiently suitable for publication.

The figures have therefore been reorganized as high-resolution composite figures rather than collections of separately compressed images. The multi-panel layout also reduces redundant plotting and allows related results to be compared directly within the same figure.

The figure captions have been rewritten so that every panel is explicitly identified as (a), (b), (c), etc., and the corresponding physical quantities and units are stated in the captions.

We believe that these changes substantially improve the readability, consistency, and publication quality of the figures.

Additional clarification regarding the scope of the manuscript

Following the reviewer's comments, we have also reconsidered the overall positioning of the manuscript.

The revised manuscript does not claim that the current three analytical models reproduce the full complexity of experimental digital holography. Instead, the study is positioned as a controlled numerical benchmark.

The main purpose is to provide:

1. analytically defined phase ground truth;

2. reproducible off-axis hologram generation;

3. controlled spectral characteristics;

4. quantitative evaluation of frequency-domain filtering;

5. quantitative evaluation of phase unwrapping; and

6. baseline error measurements for future experimental and learning-based methods.

This revised positioning is more consistent with the actual scope of the numerical experiments.

The revised manuscript explicitly acknowledges that larger, more diverse datasets and experimental validation will be necessary before the framework can be used for general-purpose deep-learning training or quantitative experimental metrology.

Summary of major revisions

In response to the reviewer, the following major changes have been made:

1. The intended role of the synthetic holograms has been clarified.

2. The manuscript no longer presents the current examples as a complete deep-learning training dataset.

3. The limitation of using only two-dimensional phase-only models has been explicitly acknowledged.

4. The absence of amplitude modulation, realistic noise, speckle, aberration, multiple scattering, and 3D structures has been discussed.

5. The unsupported quantitative claim regarding coverage of industrial and biomedical phase distributions has been removed.

6. References have been incorporated into the corresponding positions in the main text.

7. Statements requiring literature support have been revised and cited where appropriate.

8. The original large number of individual figures has been reorganized into seven multi-panel composite figures.

9. Coordinate axes, physical quantities, and units have been standardized across all figures.

10. Figure captions have been rewritten to identify individual panels and their corresponding quantities.

11. The limitations and future development of the numerical framework have been explicitly added to the manuscript.

12. The overall manuscript has been revised to present the work as a reproducible numerical benchmark rather than as a complete substitute for experimental holographic datasets.

Attachment Submitted filename: Response to the reviewers.docx

# Editor report: Decision Letter 1

**DOI:** 10.1371/journal.pone.0360375.r003

**Authors:** Maddalena Collini

16 Sep 2026

Dear Dr. shi,

The manuscript now conceptually meets the standards for publication in PLOS ONE. However, several points still require attention. In particular, the figures have not yet been properly organized: there is still considerable unused space, and there does not appear to have been a sufficient effort to optimize the arrangement of panels, for example by grouping panels with the same scale and aligning them appropriately (as sugegsted below). In addition, the mathematical expressions are still not consistently numbered and cited in the text. Finally, the code provided as supplementary material still contains characters resulting from an improper conversion, which should be corrected.

Given that these issues are primarily related to presentation and organization rather than to the conceptual content of the manuscript, the authors have the opportunity to address them. However, these points should be adequately resolved for the manuscript acceptance.

Please submit your revised manuscript by Oct 16 2026 11:59PM. If you will need more time than this to complete your revisions, please reply to this message or contact the journal office at plosone@plos.org. When you're ready to submit your revision, log on to https://www.editorialmanager.com/pone/ and select the 'Submissions Needing Revision' folder to locate your manuscript file.

- A letter that responds to each point raised by the academic editor and reviewer(s). You should upload this letter as a separate file labeled 'Response to Reviewers'.
- A marked-up copy of your manuscript that highlights changes made to the original version. You should upload this as a separate file labeled 'Revised Manuscript with Track Changes'.
- An unmarked version of your revised paper without tracked changes. You should upload this as a separate file labeled 'Manuscript'.

If you would like to make changes to your financial disclosure, please include your updated statement in your cover letter. Guidelines for resubmitting your figure files are available below the reviewer comments at the end of this letter.

If applicable, we recommend that you deposit your laboratory protocols in protocols.io to enhance the reproducibility of your results. Protocols.io assigns your protocol its own identifier (DOI) so that it can be cited independently in the future. For instructions see: https://journals.plos.org/plosone/s/submission-guidelines#loc-laboratory-protocols. Additionally, PLOS One offers an option for publishing peer-reviewed Lab Protocol articles, which describe protocols hosted on protocols.io. Read more information on sharing protocols at https://plos.org/protocols?utm_medium=editorial-email&utm_source=authorletters&utm_campaign=protocols.

As the corresponding author, your ORCID iD is verified in the submission system and will appear in the published article. PLOS supports the use of ORCID, and we encourage all coauthors to register for an ORCID iD and use it as well. Please encourage your coauthors to verify their ORCID iD within the submission system before final acceptance, as unverified ORCID iDs will not appear in the published article. *Only*  the individual author can complete the verification step; PLOS staff *cannot*  verify ORCID iDs on behalf of authors.

We look forward to receiving your revised manuscript.

Kind regards,

Maddalena Collini, PhD

Academic Editor

PLOS One

Journal Requirements:

If the reviewer comments include a recommendation to cite specific previously published works, please review and evaluate these publications to determine whether they are relevant and should be cited. There is no requirement to cite these works unless the editor has indicated otherwise.

Additional Editor Comments:

The authors have adequately addressed the reviewers’ comments concerning the conceptual aspects of the manuscript. However, a few points still need to be addressed in order to be acceptable for publication in PLOS ONE:

Figures and panel organization

The figures still need to be reorganized to meet PLOSONE standard. At present, the panels are not properly assembled, resulting in an unnecessary blank space in the lower-right portion of the figures.

• Figure 1 The panels should be arranged in six rows (as a hint), with two rows corresponding to each target type (Gaussian, four-level step, and Siemens star), while ensuring that all panels are properly aligned. For example, panels (a) and (b) share the same scale, and panel (e).a similar one Therefore, these three panels could be placed together in the first row, with the remaining three panels arranged in the second row, while maintaining consistent panel dimensions and alignment

• Figure 2: All panels share the same scale, so the scale bar can be shown on only one side. The spacing between the panels should also be reduced and kept consistent.

• Figure 3: The corresponding panels for each type of target should be aligned consistently.

• Figure 4: The panel showing the 3D error could be moved to the Supplementary Material, while the remaining panels should be grouped as suggested above.

• The same principle of consistent alignment, spacing, and grouping should be applied to the remaining figures

Mathematical expressions and parameter definitions

Each mathematical expression should be numbered and explicitly cited in the text. In addition, several parameters are introduced without adequate explanation. These should be clearly defined when they first appear. Some expression are given without comments and punctuation: for example: page 17, line 4 and 8 from the bottom.

Supplementary code: The code provided as supplementary material still contains a number of Chinese characters that were not properly translated during the conversion process. These should be translated into English and the supplementary code should be carefully checked to ensure that no untranslated characters or conversion artifacts remain.

[Note: HTML markup is below. Please do not edit.

To ensure your figures meet our technical requirements, please review our figure guidelines: https://journals.plos.org/plosone/s/figures

You may also use PLOS’s free figure tool, NAAS, to help you prepare publication quality figures: https://journals.plos.org/plosone/s/figures#loc-tools-for-figure-preparation.

NAAS will assess whether your figures meet our technical requirements by comparing each figure against our figure specifications.

# Author comment: Author response to Decision Letter 2

**DOI:** 10.1371/journal.pone.0360375.r004

22 Sep 2026

Dear Academic Editor and Reviewers,

We sincerely thank the Academic Editor and the Reviewers for their careful evaluation of our manuscript and for providing valuable comments and constructive suggestions. We have carefully considered all the comments and have made corresponding revisions to improve the clarity, consistency, presentation quality, and reproducibility of the manuscript.

In particular, we have comprehensively revised the figure layouts, standardized the mathematical expressions and parameter definitions, and carefully checked the supplementary code to eliminate untranslated Chinese characters and formatting-related errors.

Our detailed responses to each comment are provided below. All revisions have been carefully checked to ensure consistency with the requirements of the journal.

Response to the Academic Editor

Comment 1: Figure Layout and Subfigure Arrangement

Editor's Comment:

The layouts of all figures need to be further adjusted to comply with the publication standards of PLOS ONE. The current arrangement of subfigures is inappropriate, resulting in substantial unnecessary blank space, particularly in the lower-right areas of the figures. Specific requirements include reorganizing the subfigures in Figures 1–4 and applying consistent alignment, spacing, and grouping principles to all remaining figures.

Response:

Thank you for this valuable suggestion. We agree that the original figure layouts did not adequately satisfy the requirements for consistent alignment, spacing, and visual presentation.

In response, we have comprehensively revised the layouts of all figures according to the following principles:

1. Consistent subfigure dimensions: The sizes of subfigures have been standardized to ensure visual consistency throughout each composite figure.

2. Uniform alignment: Subfigures have been carefully aligned both horizontally and vertically to avoid irregular positioning.

3. Consistent spacing: The spacing between subfigures has been adjusted to maintain a uniform layout while reducing unnecessary gaps.

4. Grouping by test object: Subfigures corresponding to the same test object have been arranged according to a consistent grouping strategy.

5. Reduction of unnecessary white space: The overall figure layouts have been optimized to minimize redundant blank areas, particularly in the lower-right regions.

These revisions were implemented to improve the readability and visual consistency of the figures while better meeting the figure presentation requirements of PLOS ONE.

Comment 1.1: Figure 1

Editor's Comment:

Figure 1 may be arranged using a six-row layout, with every two rows corresponding to one type of test object: the Gaussian sample, the four-level step sample, and the Siemens star sample. All subfigures should be strictly aligned. For example, subfigures (a), (b), and (e) have the same scale, and therefore these three subfigures can be placed in the first row, with the remaining three subfigures placed in the second row. All subfigures should maintain consistent dimensions and alignment.

Response:

Thank you for providing a specific and practical suggestion regarding the arrangement of Figure 1.

We have reorganized Figure 1 according to the recommended six-row layout. The three test objects, namely the Gaussian sample, the four-level step sample, and the Siemens star sample, are now arranged using a consistent two-row structure for each test object.

During the rearrangement, we carefully considered the scale, dimensions, and spatial correspondence of the subfigures. Subfigures with consistent scale characteristics have been placed in corresponding positions, while the dimensions of all subfigures have been standardized.

Furthermore, the alignment of the subfigures has been adjusted to ensure consistent horizontal and vertical positioning. Unnecessary blank space has also been reduced to improve the overall compactness and readability of the figure.

The revised Figure 1 now follows a more consistent and structured arrangement, facilitating comparison among the three test objects.

Location of Revision: Revised Figure 1.

Comment 1.2: Figure 2

Editor's Comment:

All subfigures in Figure 2 should use the same scale. Only one subfigure needs to display the scale bar. The spacing between all subfigures should be reduced and maintained consistently.

Response:

We appreciate this suggestion regarding the standardization of scale bars and subfigure spacing.

In the revised Figure 2, all subfigures share the same scale. To avoid unnecessary repetition and visual clutter, the scale bar is retained in only one representative subfigure, while the other subfigures use the same scale reference.

In addition, the spacing between adjacent subfigures has been reduced and standardized. The margins surrounding the composite figure have also been adjusted to minimize unnecessary blank space while maintaining sufficient separation between individual subfigures.

These modifications improve the visual consistency of Figure 2 and facilitate direct comparison among the different test objects.

Location of Revision: Revised Figure 2.

Comment 1.3: Figure 3

Editor's Comment:

The alignment logic for subfigures corresponding to each test object in Figure 3 should be fully consistent with that used for other subfigures of the same type.

Response:

Thank you for this comment.

We have revised Figure 3 to ensure that the alignment of subfigures corresponding to each test object follows a consistent layout principle. Subfigures representing the same type of test object are now arranged using standardized dimensions, alignment positions, and inter-subfigure spacing.

The arrangement has been reviewed to ensure that the corresponding subfigures of the Gaussian sample, four-level step sample, and Siemens star sample follow the same organizational logic. This revision improves the consistency of the figure structure and makes comparisons between different test objects more straightforward.

Location of Revision: Revised Figure 3.

Comment 1.4: Figure 4

Editor's Comment:

The subfigures presenting the 3D error results may be moved to the supplementary materials. The remaining subfigures should be reorganized according to the unified layout standards described above.

Response:

We thank the Editor for this suggestion.

Following this recommendation, we have reconsidered the organization of the 3D error visualization results. The relevant 3D error subfigures have been separated from the main figure and incorporated into the supplementary materials, thereby reducing the visual complexity of the main text.

The remaining subfigures in Figure 4 have been reorganized according to the unified layout principles applied throughout the manuscript, including consistent subfigure dimensions, alignment, spacing, and grouping of the same type of test object.

This revision allows the main figure to focus on the essential reconstruction and error analysis results while retaining the detailed 3D error visualizations in the supplementary materials.

Location of Revision: Revised Figure 4 and Supplementary Materials.

Comment 1.5: Remaining Figures

Editor's Comment:

All remaining figures should follow the core principles of consistent alignment, uniform spacing, and grouping of subfigures according to their type.

Response:

We fully agree with this recommendation.

In addition to Figures 1–4, we have reviewed and revised the layouts of all remaining figures in the manuscript. The following standards have been applied consistently:

• Uniform subfigure dimensions.

• Consistent horizontal and vertical alignment.

• Uniform spacing between adjacent subfigures.

• Grouping of subfigures according to the corresponding test object or analysis category.

• Reduction of unnecessary margins and blank areas.

• Consistent placement of labels, scale bars, and other graphical annotations.

A comprehensive visual inspection was conducted to identify and correct inconsistencies in the arrangement of subfigures.

These revisions improve the overall presentation quality and consistency of the manuscript figures.

Location of Revision: All revised figures in the manuscript and supplementary materials.

Comment 2: Mathematical Formulas and Parameter Definitions

Editor's Comment:

All mathematical expressions should be numbered consistently, and the corresponding equation numbers should be explicitly cited in the main text. Some parameters are insufficiently explained when first introduced and should be clearly and completely defined at their first occurrence. In addition, certain mathematical expressions lack accompanying explanations and punctuation marks, such as those appearing on page 17, lines 4 and 8 from the bottom.

Response:

We sincerely thank the Editor for identifying these issues concerning mathematical notation, parameter definitions, and the presentation of equations.

We have systematically reviewed the mathematical expressions throughout the manuscript and made the following revisions.

2.1 Standardization of Equation Numbering

All major mathematical expressions requiring independent reference have been assigned consistent equation numbers.

The corresponding equation numbers have also been explicitly cited in the main text wherever the equations are introduced, explained, or subsequently referenced. This ensures a clear connection between the mathematical expressions and their associated descriptions.

2.2 Improvement of Parameter Definitions

We have carefully checked the first occurrence of each important parameter in the manuscript.

Where necessary, additional explanations have been provided to clarify:

• The physical meaning of each parameter.

• The corresponding units.

• The role of each parameter in the mathematical model.

• The relationship between the parameter and the experimental or simulation setup.

These definitions have been added at the first relevant occurrence of each parameter to improve the clarity and reproducibility of the proposed method.

2.3 Revision of Mathematical Expressions and Punctuation

We have revised the mathematical expressions identified by the Editor, including those located on page 17, fourth and eighth lines from the bottom.

The associated explanatory text and punctuation have been supplemented to ensure that each mathematical expression is properly integrated into the surrounding paragraph. We have also reviewed the consistency of mathematical symbols, notation, and explanatory statements throughout the manuscript.

These revisions improve the readability, logical continuity, and formal presentation of the mathematical content.

Location of Revision: Relevant mathematical expressions and parameter definitions throughout the manuscript, including the specified section on page 17.

Comment 3: Supplementary Code Formatting and Untranslated Characters

Editor's Comment:

The code file submitted as supplementary material still contains a considerable number of Chinese characters that were not correctly translated during the formatting conversion process. All such characters should be replaced with English, and the entire supplementary code file should be checked line by line to ensure that no untranslated Chinese characters, garbled text, or formatting-related abnormal symbols remain.

Response:

Thank you for pointing out this issue in the supplementary code.

We recognize that untranslated Chinese characters and formatting-related inconsistencies may affect the readability, accessibility, and reproducibility of the supplementary material, particularly for an international readership.

In response, we have conducted a comprehensive review of the supplementary code file. The following revisions have been performed:

1. Translation of Chinese text: All Chinese comments, descriptions, labels, and other text within the code have been translated into English.

2. Removal of abnormal characters: Formatting-related garbled characters and abnormal symbols introduced during the document conversion process have been identified and corrected.

3. Line-by-line inspection: The supplementary code has been reviewed line by line to identify any remaining untranslated text or formatting inconsistencies.

4. Consistency of code annotations: Comments, variable descriptions, and explanatory statements have been checked to ensure consistent English terminology.

5. Code integrity check: The revised code has been reviewed to ensure that the text-formatting modifications do not unintentionally affect the structure or execution of the original code.

We have also performed a final inspection of the supplementary code to minimize the possibility of residual untranslated characters or conversion-related formatting errors.

The revised supplementary code has been prepared for resubmission together with the revised manuscript.

Location of Revision: Revised Supplementary Code File.

Concluding Statement

We sincerely appreciate the Academic Editor's and Reviewers' careful assessment of our manuscript and their constructive recommendations.

In response to the comments, we have revised the figure layouts, standardized the mathematical expressions and parameter definitions, and comprehensively checked the supplementary code. These revisions were undertaken to improve the clarity, consistency, presentation quality, and reproducibility of the manuscript.

We believe that the revised manuscript and supplementary materials have been substantially improved as a result of these changes. We hope that the revisions adequately address the concerns raised during the review process.

Thank you again for your time and consideration.

Attachment Submitted filename: Response to the reviewers.doc

# Editor report: Decision Letter 2

**DOI:** 10.1371/journal.pone.0360375.r005

**Authors:** Maddalena Collini

27 Sep 2026

Controlled Numerical Generation and Reconstruction of Off-Axis Digital Holograms with Object-Specific Frequency-Domain Filtering

PONE-D-26-30391R2

Dear Dr. shi,

We’re pleased to inform you that your manuscript has been judged scientifically suitable for publication and will be formally accepted for publication once it meets all outstanding technical requirements.

Within one week, you’ll receive an e-mail detailing the required amendments. When these have been addressed, you’ll receive a formal acceptance letter and your manuscript will be scheduled for publication.

An invoice will be generated when your article is formally accepted. Please note, if your institution has a publishing partnership with PLOS and your article meets the relevant criteria, all or part of your publication costs will be covered. Please make sure your user information is up-to-date by logging into Editorial Manager at Editorial Manager® and clicking the ‘Update My Information' link at the top of the page. For questions related to billing, please contact billing support.

If your institution or institutions have a press office, please notify them about your upcoming paper to help maximize its impact. If they’ll be preparing press materials, please inform our press team as soon as possible -- no later than 48 hours after receiving the formal acceptance. Your manuscript will remain under strict press embargo until 2 pm Eastern Time on the date of publication. For more information, please contact onepress@plos.org.

Kind regards,

Maddalena Collini, PhD

Academic Editor

PLOS One

Additional Editor Comments :

Please note that Figure 6 was truncated when the file was generated. Kindly check and correct Figure 6 in the revised proofs.

# Editor report: Acceptance letter

**DOI:** 10.1371/journal.pone.0360375.r006

**Authors:** Maddalena Collini

PONE-D-26-30391R2

PLOS One

Dear Dr. Shi,

I'm pleased to inform you that your manuscript has been deemed suitable for publication in PLOS One. Congratulations! Your manuscript is now being handed over to our production team.

At this stage, our production department will prepare your paper for publication. This includes ensuring the following:

* All references, tables, and figures are properly cited

* All relevant supporting information is included in the manuscript submission,

* There are no issues that prevent the paper from being properly typeset

You will receive further instructions from the production team, including instructions on how to review your proof when it is ready. Please keep in mind that we are working through a large volume of accepted articles, so please give us a few days to review your paper and let you know the next and final steps.

Lastly, if your institution or institutions have a press office, please let them know about your upcoming paper now to help maximize its impact. If they'll be preparing press materials, please inform our press team within the next 48 hours. Your manuscript will remain under strict press embargo until 2 pm Eastern Time on the date of publication. For more information, please contact onepress@plos.org.

You will receive an invoice from PLOS for your publication fee after your manuscript has reached the completed accept phase. If you receive an email requesting payment before acceptance or for any other service, this may be a phishing scheme. Learn how to identify phishing emails and protect your accounts at https://explore.plos.org/phishing.

If we can help with anything else, please email us at customercare@plos.org.

Thank you for submitting your work to PLOS One and supporting open access.

Kind regards,

PLOS One Editorial Office Staff

on behalf of

Dr. Maddalena Collini

Academic Editor

PLOS One
